# Function, life histories, and biographies of Lower Paleolithic patinated flint tools from Late Acheulian Revadim, Israel

**DOI:** 10.1038/s41598-022-06823-2

**Published:** 2022-03-03

**Authors:** Bar Efrati, Ran Barkai, Stella Nunziante Cesaro, Flavia Venditti

**Affiliations:** 1 Department of Archaeology and Ancient Near East Cultures, Tel Aviv University, POB 39040, 66978 Tel Aviv, Israel; 2 Scientific Methodologies Applied To Cultural Heritage (SMATCH), Rome, Italy; 3 Department of Early Prehistory and Quaternary Ecology, University of Tübingen, Schloß Hohentübingen, Burgsteige 11, 72070 Tübingen, Germany; 4 LTFAPA Laboratory, Department of Classics, Sapienza University of Rome, P.Le Aldo Moro, 00185 Rome, Italy

**Keywords:** Archaeology, Archaeology

## Abstract

Flint tools exhibiting modified patinated surfaces (“double patina”, or post-patination flaked items) provide a glimpse into Paleolithic lithic recycling, stone economy, and human choices. Different life cycles of such items are visually evident by the presence of fresh new modified surfaces alongside old patinated ones (according to color and texture differences). New modifications testify to a gap in time between the previous life cycle of the patinated flaked item and its new one. The aim of the current study is to reconstruct the functional properties and life cycles of a sample of modified patinated flaked tools from Late Acheulian Revadim, Israel by applying use-wear and residue analyses. The results of the functional study allow a better understanding of the practical reasoning behind the collection and recycling of old flint tools, while additional inputs from theoretical and methodological advancements assist in reconstructing their probable role in the worldviews of the site’s inhabitants.

## Introduction

Patina is a chemical alteration that occurs over time, and under certain environmental and chemical conditions, on the surfaces of numerous types of materials (e.g., various stone types, in particular siliceous stones, as well as on metals and glass). Here we focus on patina of flint^1–3^. Scholars distinguish between various types of patina of flint, each resulting from a combination of different factors related to the structure (and microstructure) of the flint and its type; biogenic factors; and environmental factors^1,2,4–12^. However, despite these variations, types of patina are primarily distinguished by color differences and the roughness of the material as compared to “fresh” flint^4,13^. Well-developed patinas can be distinguished by the naked eye, and “fresh” unpatinated surfaces, or surfaces covered in a different type of patina, can be discerned by color, gloss, and texture.

Of the various available subjects of study and terminology^1–3,5–8,10,14–20^, double patinated items are studied in current archaeological research mostly in the context of lithic recycling. Recycled items made from ‘older’ patinated flaked items have been documented at many Early to Upper Paleolithic sites in the Levant and beyond^21–34^, as well as at sites dated to later periods^35–44^.

At the Late Acheulian site of Revadim, Israel, flaked patinated items were collected and recycled in significant numbers and used alongside fresh flint and other technological trajectories characterized as recycling (mainly the production and use of small flakes; (Supplementary Fig. S2)^32,45–47^. Revadim is an open-air site dated to ca 500–300 kyr^48,49^ (Supplementary Fig. S1, and supplementary information S1). Based on the lithic assemblages, the significant handaxes component, the presence of straight-tusked elephants, and given the preliminary radiometric dates, the entire anthropogenic occupation was assigned to the Late Acheulian cultural-complex of the Levant^48–52^.

Flaked patinated items from Revadim were collected in significant numbers by the site’s inhabitants and recycled into new tools (for a detailed explanation see supplementary information S1). These modified flint items thus exhibit two life cycles, with a clear time gap between them. In its first life cycle, such an item was detached from the original nodule/core and then shaped into a tool. It was eventually discarded, and its surface became patinated over time. Its second life cycle began when someone collected the flaked patinated item for further shaping/flaking, exposing the fresh flint surface underneath the patinated one. This item, too, was eventually discarded. The time gap between the two cycles is discernable in the patinated flaked surfaces themselves and in the removal of parts of these surfaces when the tools were reshaped. These clearly visible markers identify these items as the products of recycling by later humans^15,23,25^. The items could have been collected during recurrent and/or extended visits to the same locality, brought to the site from elsewhere during excursions, and/or selected from the arsenal of old flaked flint items available on-site from prior activities. Regardless of exactly who collected them and under what circumstances, we contend that their selection, collection, and recycling were intentional, conscious, and carried out regularly by early humans during Lower Paleolithic times^34,53,54^. We further assume that the collectors were motivated by a combination of practical and perceptual reasons, and not solely by straightforward economic constraints^55–58^.

We conducted a functional study of these "double patina”, or post-patination flaked tools, integrating use-wear and residue analysis to reconstruct their functional life cycles. Items with natural surfaces covered in patina or flaked patinated items that show no evidence of new modified surfaces will not be dealt with here. Henceforth, we will use the term ‘post-patination flaked items’ (PPF items) instead of ‘double patina’ to describe these items^27,34^. Our goal here is fourfold: (1) to study the function of PPF tools in the course of their second life cycle (post recycling); (2) to look for indications regarding their original use, during their first (pre-patination) life cycle; (3) to check whether their first and second life cycles can be reliably distinguished. Finally, the functional analysis will be accompanied by (4) a discussion of the possible reasoning behind the collection and use of flaked patinated items and the seemingly intentional preservation of the original patinated surfaces. Theoretical grounds for our hypothesis will accompany and complement the functional characterization of the items’ biographies in order to place the phenomenon in a broader perspective. In doing so, we apply an integrated and multidisciplinary methodology that combines: (1) different analytical techniques for describing and interpreting use-wear traces and use-related residues on stone tools; and (2) recent theoretical and methodological advancements for reconstructing the probable role of these assumed mnemonic items in the worldviews of the site’s inhabitants. Our hypothesis is that the collected items’ characteristics were intentionally preserved; in order to maintain the desired properties of the functional tool, to preserve the item’s biographies, and to serve as an item of memory to a site occupied throughout time^58–69^.

**Figure 1:**
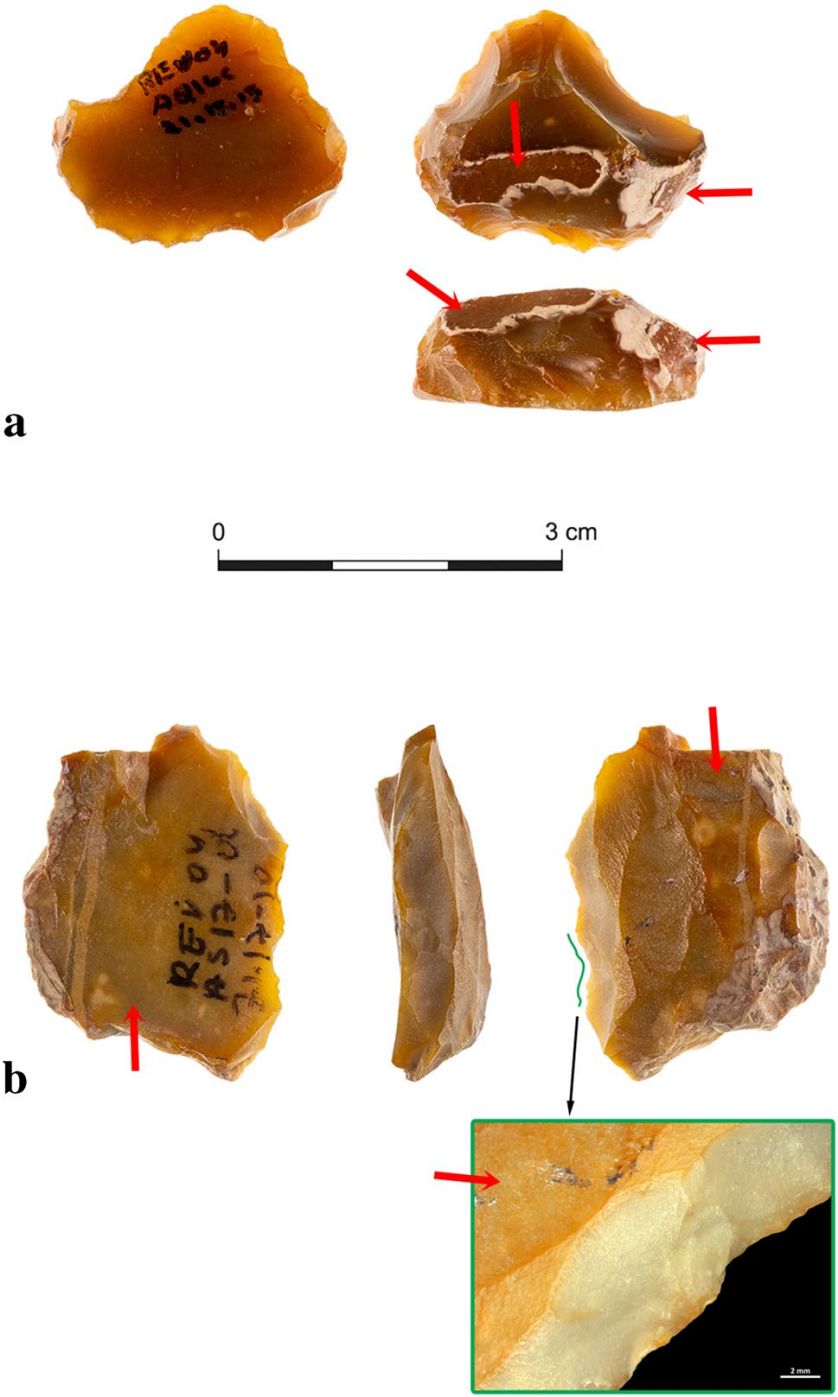
The red arrows point for the patinated areas. (**a**) Post-patination flaked scraper from Revadim (layer C3). Remains of a previous patinated flaked surface can be observed only on the dorsal face of the item, while its ventral face and retouch are newer, and expose the natural color of the flint. (**b**) Post-patination flaked shaped item from Revadim (layer C3). The yellow-orange areas are the old patinated flaked surfaces, while the new modifications created a thin, translucent, denticulated edge visible on the left and right images and appearing as grey scars on the middle image. In the case of this item, the morphology and colors of the old blank are almost fully preserved, while the recycling modification is minimal and specific.

The current study discusses a sample of 49 ‘old’ fully patinated items that have served as blanks for the making of ‘new’ tools, thus exhibiting flaked patinated ‘old’ surfaces with minimal and specific new retouch that exposes the surfaces of the fresh flint underneath (Fig. 1) from Revadim layer C3 (see supplementary information S1).

## Results

The surfaces of the sampled items exhibited different degrees of preservation according to their older or newer modifications (Supplementary Fig. S3). A medium-to-high degree of rounding and polishing was recorded at low and high magnifications on the older active edges, as well as on the majority of the surface, thus corresponding to the first life cycle (Supplementary Fig. S3a). In this case, functional reconstructions are based on edge removals and limited to the interpretation of motion and hardness of materials processed.

The new modified edges showed rather less weathered surfaces at low magnification, and relatively sharp outlines. At high magnification, most of the new edges showed medium-to-covering polished surfaces, thus confirming the trend already observed in layer C3^47^. On a few cases, surfaces on the new modified edges exhibited less patinated surfaces and final interpretations relied on the macro and micro wear observations (Supplementary Fig. S3b).

All in all, given the absence of strong mechanical post-depositional stress (e.g., heavy rolling, post-depositional edge scarring, fractures), the sample was considered suitable for functional investigation if edge removals represented by use-related scars on the active edge of the tools, and eventually polish, could be identified.

### Use-wear results

Out of the 49 items selected for this study, 28 bear use-wear traces related to their first (n = 3), second (n = 16), or both use-cycles (n = 9), accounting for 57% of the total sample (Supplementary Table S1).

#### First life cycle

The degree of preservation of the patinated surfaces allowed the pre-patina functional reconstruction of 12 out of the 28 tools that we identified as used. We interpreted six of the remaining tools as non-diagnostic for a reliable functional reconstruction of their first life-cycle, while eight others showed no evidence of use on their old edges. In the case of the remaining three items, the older patinated active edge was later cut by the new modification, leaving only a small portion of the original edge bearing damage patterns but with no clear functional evidence. We thus considered the original function of these artifacts as indeterminable (Supplementary Table S1).

**Figure 2:**
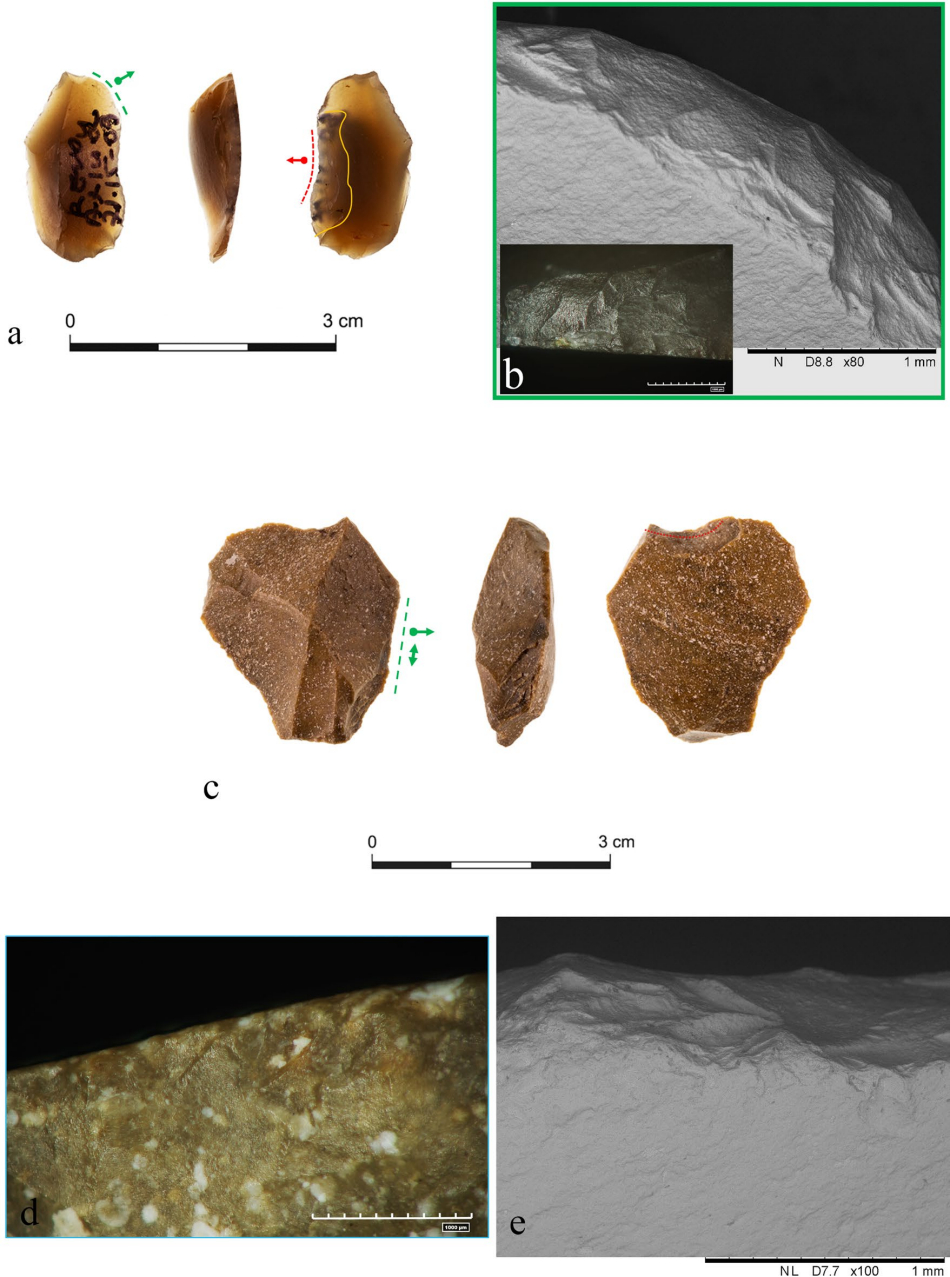
(**a**) Item #7 (retouched special spall, Ax14a 71.15-09) showing two active edges, one from the first use cycle (green dotted line) and one from the second and last use cycle (red dotted line); (**b**) SEM-micro graph of the steep edge created by the retouch of the proximal edge of item #7; (**c**) item #17 (retouched flake, Av14c 71.07-06) showing evidence of mixed activity along its old edge (green dotted line), and no evidence of use with the new edge created by the last modification (red dotted line); (**d**) step scars along the old active edge of item #17; (**e**) SEM-micro graph of the old active edge of item #17, showing use related scarring in combination with a medium to high degree of rounding of the old active edge.

The used first-cycle edges were either retouched or not, according to the type of activity performed. We noticed, for example, that transversal motions intended as scraping activities were carried out with retouched edges aimed at creating a steep front (green dotted line in Fig. 2a,b).

Use-wear evidence show that five tools were used to cut soft and medium materials during their first life-cycle (one side scraper, one notch, one retouched flake, one retouched broken flake, and one varia tool), three tools were used to scrape soft-medium and medium to hard materials (one side scraper, one retouched special spall, and one retouched flake), one retouched flake was used for mixed activities on a soft-medium material (Fig. 2c–e), and the chopping tool was used for chopping activities. The remaining three tools were possibly used for activities with longitudinal motions; however, the degree of patination of their older edges precluded further results.

#### Second life cycle

**Figure 3:**
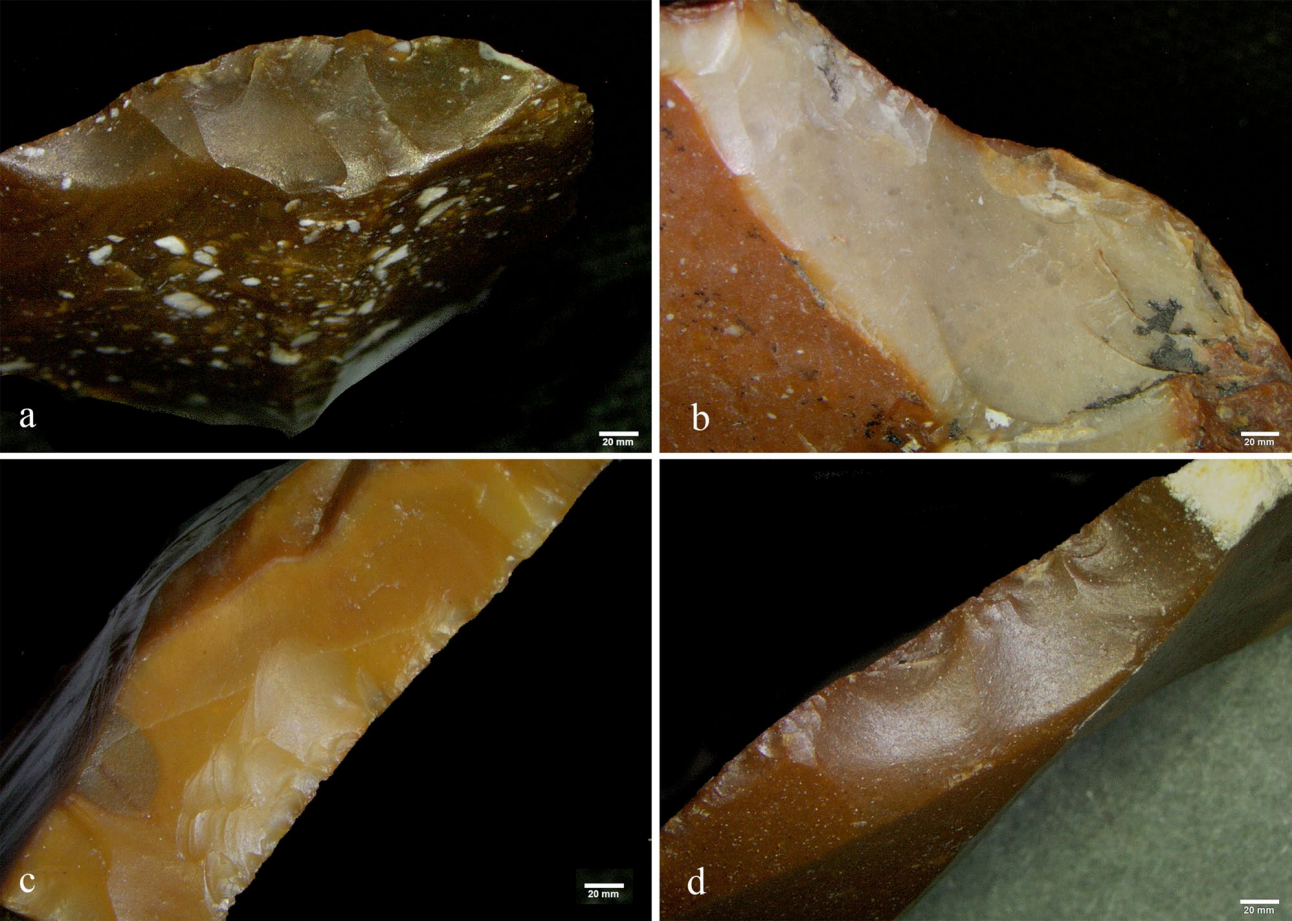
The different minimal shaping methods used for the creation of the new edges. (**a**) Retouched broken flake (#26 Av15a 71.11-08); (**b**) Varia tool (#21 Au13c 71.17-14); (**c**) Notch (#30 Av13a 71.18-14); (**d**) Notch (#13 Av14a 71.13-10).

We first determined the new active edges of the tools by observing the differences in patination between the new modifications and the rest of the surface. The tools were then observed again to determine whether the scars on the modified edges were the result of deliberate edge removals/retouch or the result of use-wear or post-depositional processes. The temporal relations between the scars on each new active edge were also examined^70^ (Fig. 3). The high degree of preservation allowed the post-patina functional interpretation of 25 out of the 28 used tools (Supplementary Table S1). Most of these tools (n = 16) bear one new active edge that is not related to the first life cycle of the item. The remaining nine tools demonstrate two (in one case even three) new potential active edges shaped along the outline of the patinated artifacts. However, we identified only one edge as used in all these nine cases.

Further macroscopic observations allowed us to distinguish between at least two technological manufacturing procedures in creating the new edges among the 28 tools bearing evidence of use: first, in most cases (n = 21), the new edge was created by modifying the patinated old edge using retouch, without previous modifications (Fig. 3c,d). In seven other cases a fresh surface was prepared prior to the subsequent retouch, in order to first create the desired morphological edge profile of the new active edge (e.g., a removal of parts of the old patinated surface where the desired edge was to be placed) (Fig. 3a,b). Only one item (a retouched broken flake), demonstrating two new edges, presents a combination of these two procedures. Most of the new modified edges were used for transversal motions performed while processing different materials. Many of the new edges that were associated with the performance of transversal motions showed a tendency toward concave shaping. Two tools displayed no evidence of use on their new edges, and two additional new edges showed unreliable evidence of use.

**Figure 4:**
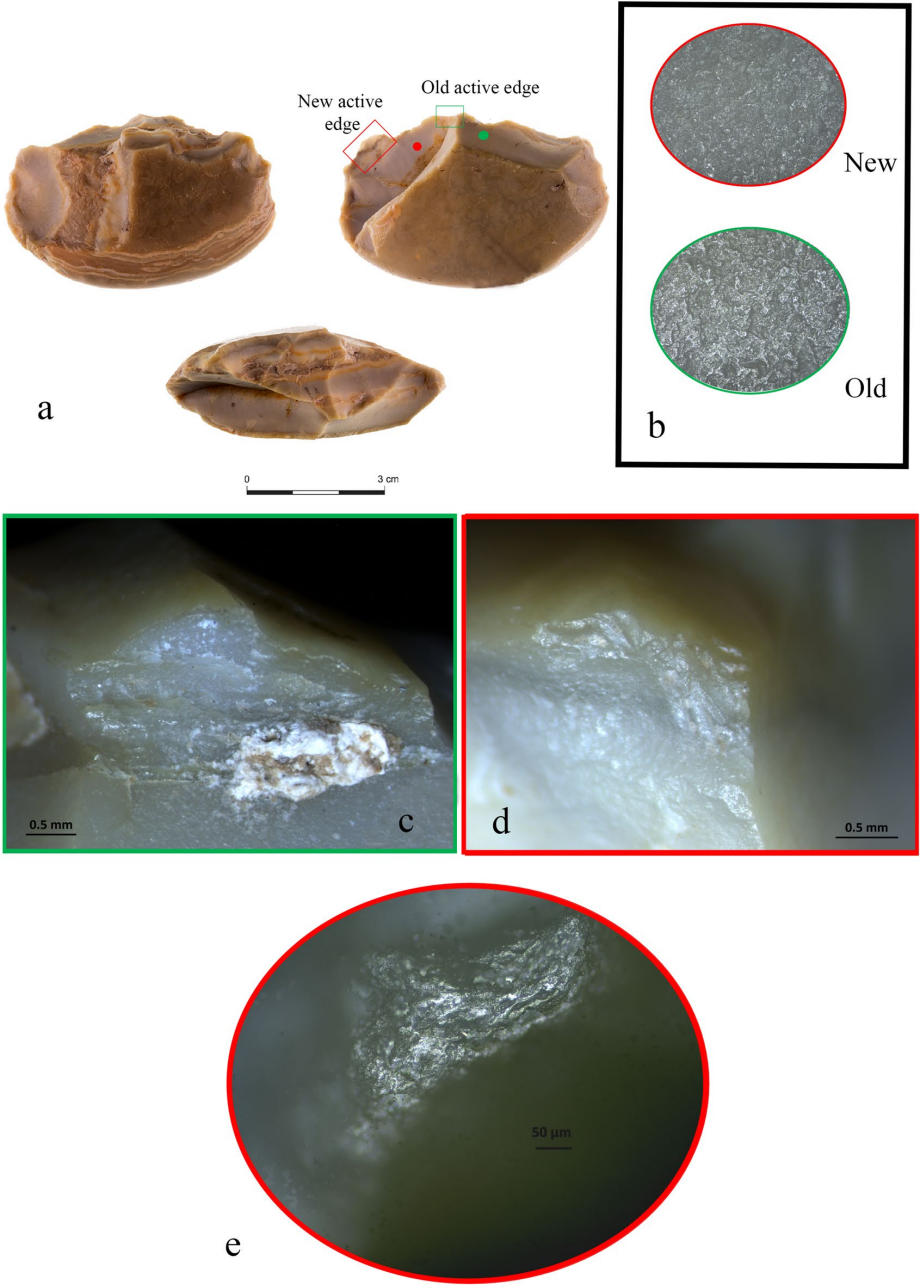
(**a**) Chopping tool (#49 Aq17c 71.03-70.01); (**b**) patination differences between the old and new exposed surfaces of the chopping tool; (**c**) production scars bearing calcite accumulation at the intersection between the item’s old and new active edges (green square); (**d**) edge removals and scarring related to chopping along the item’s new active edge (red square); (**e**) bone-line polish observed in association with the edge removals found along the item’s new active edge.

Edge removals characterization indicated that 16 tools were used for scraping activities on soft-medium and medium materials (seven side scrapers, five notches, two denticulates, one retouched flake, and one varia tool), while five tools were used for scraping activities on medium-hard materials (one notch, two retouched flakes, one retouched broken flake, and one varia tool). Two tools were used to perform a combination of transversal and longitudinal motions (one retouched special spall, and one retouched broken flake), and the chopping tool was used to chop bones, based on the combination of polish and edge removals characterization (see below; Fig. 4).

In two cases, better preservation of the lithic micro-surface allowed us to characterize the nature of the worked material. The single chopping tool in the sample (#49 AQ17c71.03-70.01) show pre- and post-recycling evidence of use (Fig. 4a). Its two cycles are easily discernable by the difference in color and texture between the old patinated and the new fresh surfaces (Fig. 4b). We found that the chopping tool was possibly used to chop a hard material during its first use-cycle (Fig. 4c), but its edge removals overlaps a series of stepped technological scars, thus preventing conclusive interpretation. The old active edge is also cut by a later removal of a large flake that occurred during the second and last stage of its modification (recycling). This removal created a pointed edge extremity that was used to chop bones. The last use of the chopping tool is more clearly identifiable, due to a well-preserved but localized smooth and domed bone-like polish associated with crush marks bearing step-terminations (Fig. 4d,e).

**Figure 5:**
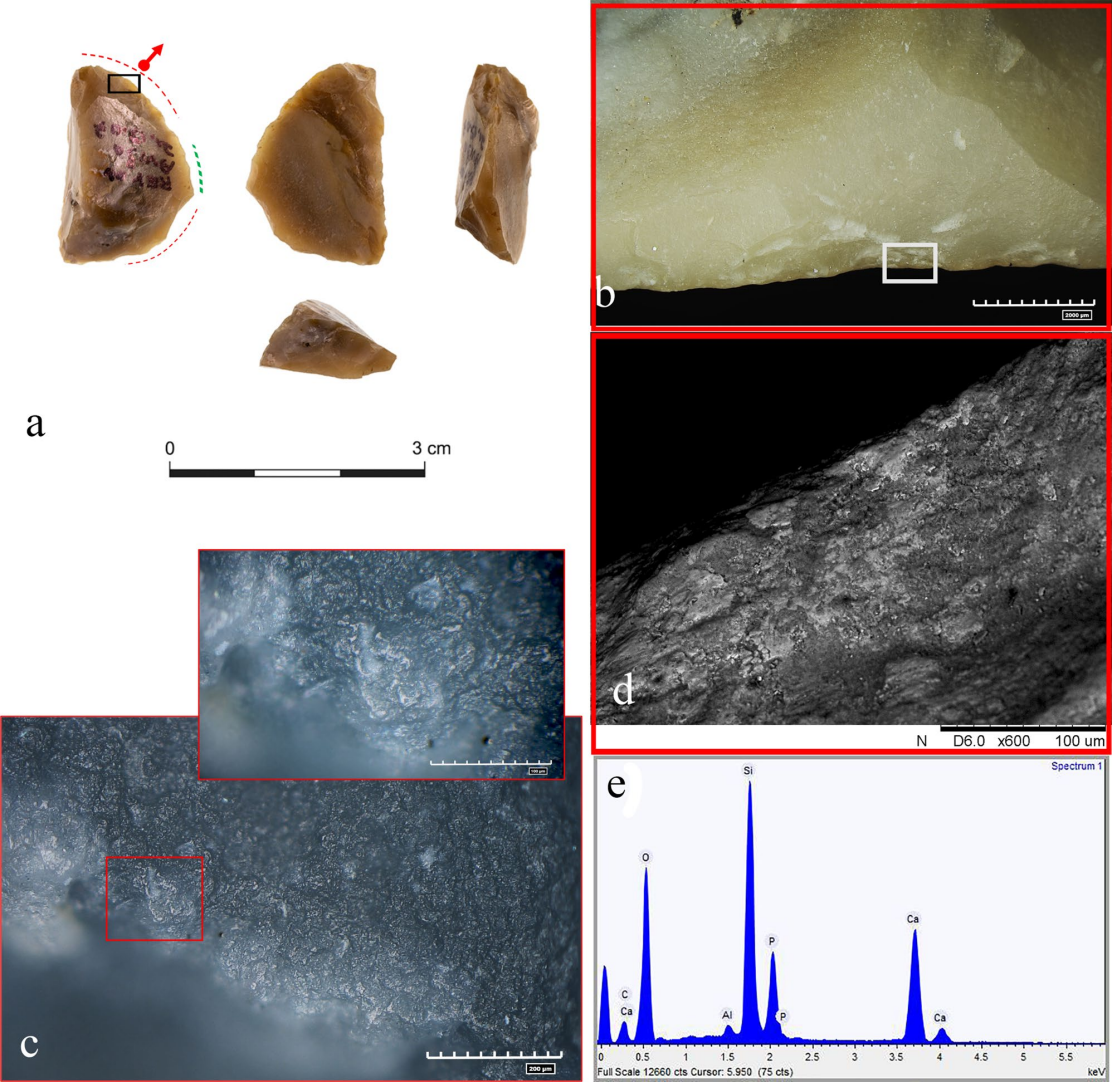
(**a**) Retouched flake (#22, Av13a 71.13-07) showing new proximal and distal active edges (red dotted lines), and a small portion of the tool’s original active edge (green dotted line); (**b**) edge removals with bone residues (white square); (**c**) micro polish along the new distal active edge, and a related close-up view; (**d**) SEM-micro graph on a portion of the new active edge with bone micro residues; (**e**) elemental composition of the bone micro-residue.

In the second case, one retouched flake (#22 AV13a71.13-07) shows new proximal and distal modifications, extending all along the convex outline of the tool (red dotted line), eventually leaving only a small portion of the original edge still in place (green dotted line) (Fig. 5a). The remaining old edge shows no clear use-related scars, while among the two new distal and proximal modifications only the proximal one exhibits evidence of use. A series of step and hinge scars, with a close regular distribution, are particularly noticeable—located on the dorsal face of the item, where a continuous rough to smooth polish with a domed topography developed on the protruding points of the flint texture (Fig. 5b,c). The observation was validated with a scanning electron microscope, which highlighted the presence of bright compacted patches of micro-remains spread inside a use-related scar along the dorsal active edge (Fig. 5d). The elemental composition, which shows the proper ratio of calcium and phosphorous, coupled with the item’s topographical features, confirmed the presence of bone micro-remains in the form of hydroxyapatite (Fig. 5e). The tool was interpreted to have been used on fleshy tissues, including contact with bone, probably during butchery activity.

### Micro-residue characterization and interpretation—the first and second life cycles

Given the positive results obtained in past residue analyses on artifacts from Area C3^47,71,86^, we paid particular attention to the detection of micro-residues resulting from the contact of the tools with the worked materials. We considered use-related residues as reliable only when found strongly adhering to the tool’s surface and when a strict correlation with the use-wear traces was shown. We recognized residue of modern contamination on a few items (e.g., human skin flakes and modern fibers) however, they did not adhere to the tools, had a random distribution, and did not show any correspondence with the use-wear traces (see below).

#### Morphological analysis

**Figure 6:**
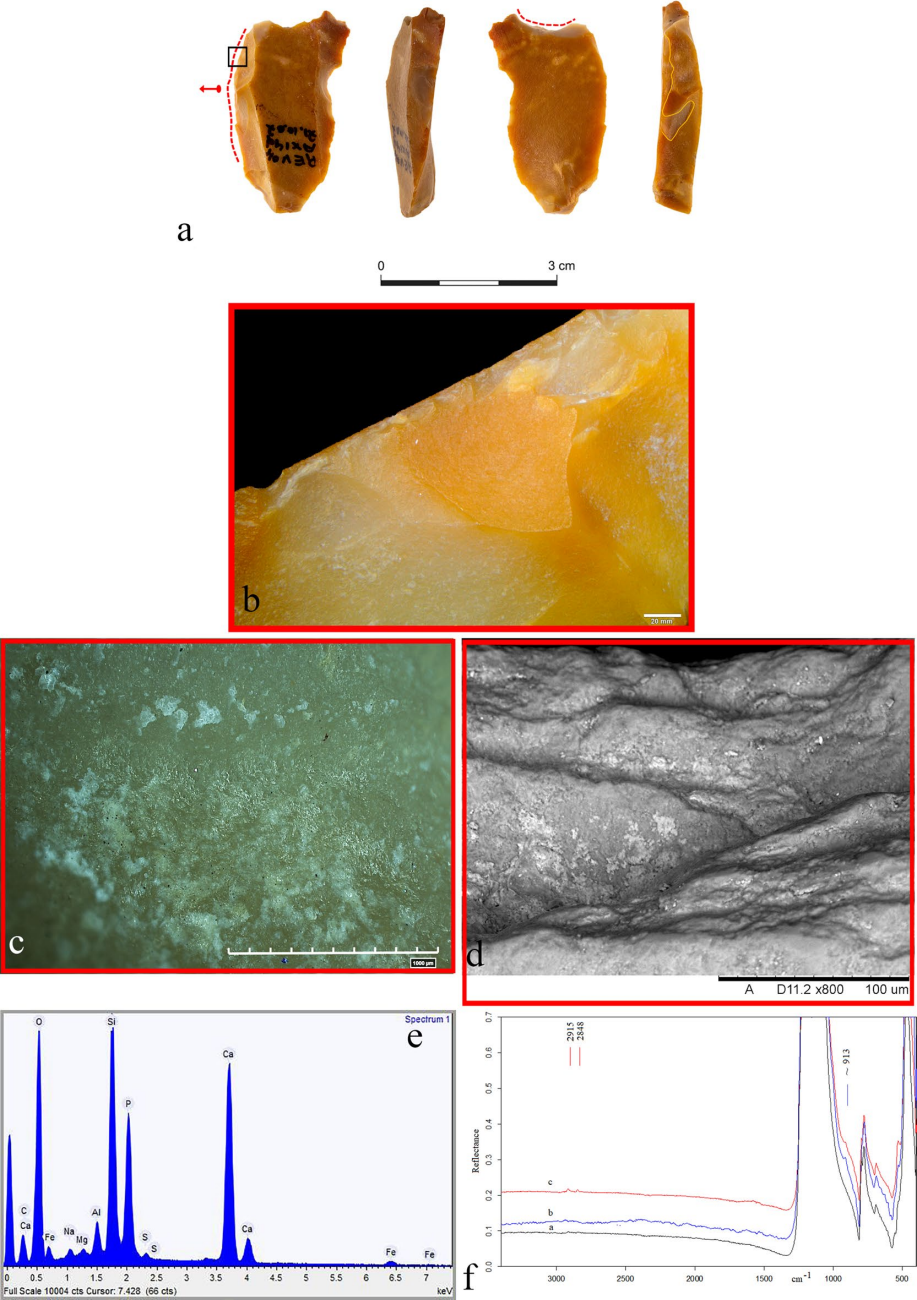
(**a**) Notch (#11 Ax14d 71.10-07) showing two new modified active edges (red dotted lines). The black square marks the location where bone residues were detected. The yellow line marks the delineation of the new modification; (**b**) edge removals along one of the item’s new edges; (**c**) bone micro-residues along one of the item’s new edges; (**d**) SEM-micro graph showing bone micro-residues entrapped in the use-related scars (notice the high degree of rounding); (**e**) elemental analysis of bone micro-residues; (**f**) micro-FTIR spectra showing: (**a**) a spot without residue measured on the archaeological tool, (**b**) micro-residues of hydroxyapatite on an experimental tool used for removing the periosteum from a fresh bone, (**c**) presence of micro-residues of hydroxyapatite detected along the new active edge of the archaeological tool.

**Figure 7:**
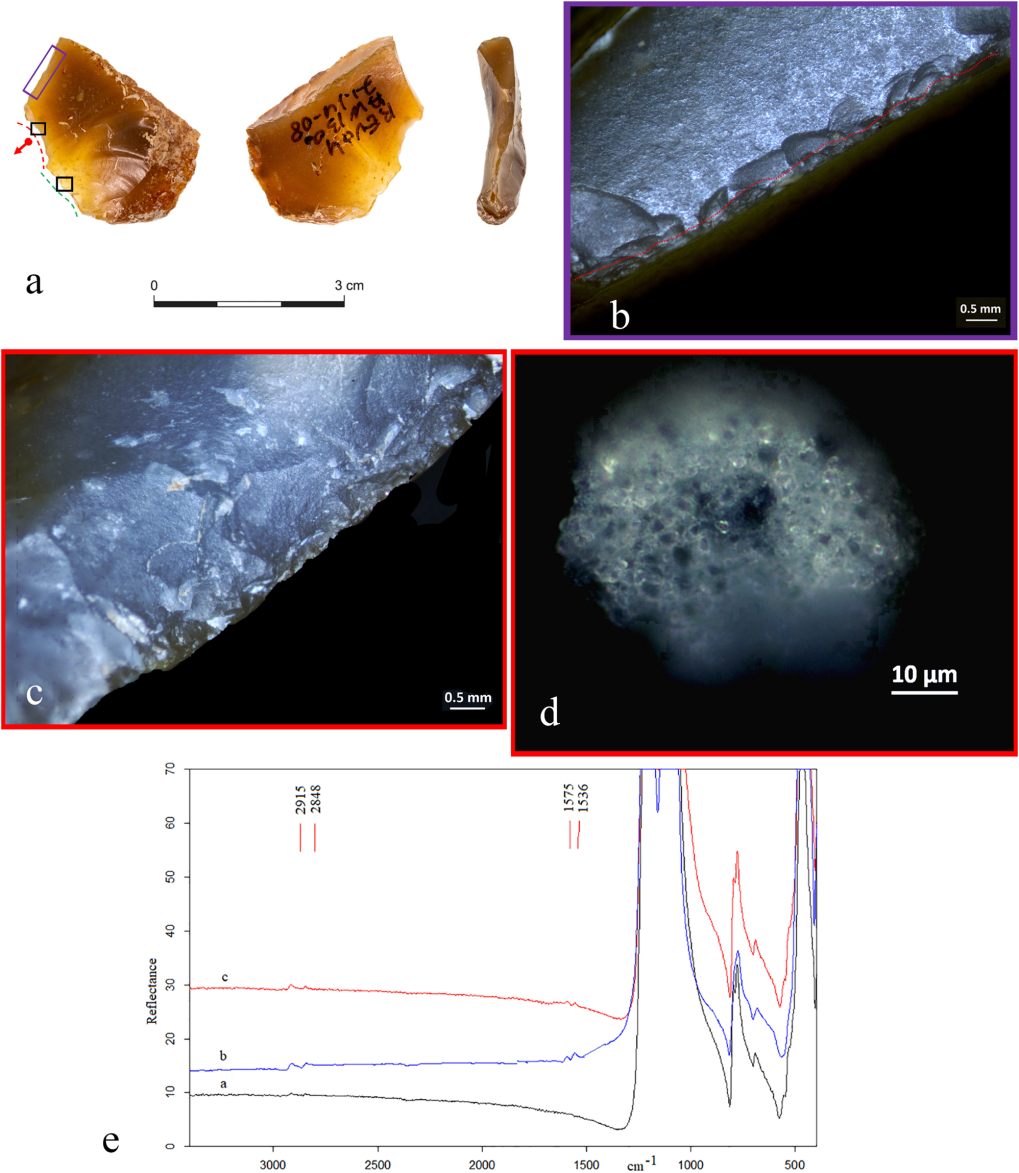
(**a**) Retouched flake (#35, Aw13d 71.14-08) showing the remaining part of the old active edge (green dotted line) that was cut by the new, and last, active edge (red dotted line). The purple square marks the possible area of prehension. The black squares mark the locations where adipocere and fat droplets were detected; (**b**) retouch evident on the item’s distal edge, showing overlapping rounded scars with a close and regular distribution interpreted as possible traces of prehension; (**c**) edge damage along the item’s new active edge; (**d**) circular accumulation of superimposed bluish birefringence fat droplets along the item’s active edge; (**e**) micro-FTIR spectra showing: (**a**) a spot without residue measured on the archaeological tool (sample control), (**b**) micro-residues of adipocere on a experimental tool used during butchery activity, (**c**) presence of micro residues of adipocere detected below the new active edge of the archaeological tool.

The entire sample of 49 tools was morphologically examined for residues during use-wear analysis at low and high magnification. Macro-residues were identified on eight tools using optical microscopes. Six of these presented micro-residues located along their newer active edge (Supplementary Table S2). Seven tools exhibited amorphous patches of whitish and whitish/yellowish deposits, smeared below and along their active edges and associated with the use-related scars (Figs. 5d, 6a–c; Supplementary Fig. S7c–e). Under the scanning electron microscope they appear with light and bright tonalities, as well as with a flat and smooth topography. In rare cases, where the deposits are thicker, they exhibited a mud-cracked appearance. On the eighth tool, a mass of birefringent fat droplets was instead observed with a circular polarized light under the metallurgical microscope. The mass was located inside the new use-related retouch scars of one of the retouched flakes (Fig. 7a–d).

#### Spectroscopic analyses

To ensure reliable interpretation of the recorded macro-residues on the 8 retouched tools, we complemented the morphological analysis with EDX and FTIR. An additional 14 artifacts showing the best evidence of use but exhibiting no visible macro residues were also chemically and elementally inspected for a more comprehensive residue analysis.

The amorphous whitish patches diagnosed on seven tools were first examined using EDX analysis to determine their elemental composition. We found they were all composed of calcium and phosphorus at a ratio of approximately 2:1 (Fig. 6d–e; Supplementary Fig. S9f). Calcium and phosphorus are the two main components of the mineral part of bone (i.e., hydroxyapatite), and when the two elements exhibit a well-established and specific ratio, as in the case of the seven tools, the residues are considered the fingerprint of bone remains^47,71–74^ (see Supplementary Information S1 for more details).

The same residues were subsequently cross-checked with infra-red spectroscopy. Four out of the seven tools exhibited a shoulder at ~ 913 cm^−1^, thus corresponding to the P=O stretching mode of the PO_4_^3−^ group of hydroxyapatites (Ca_5_(PO_4_)_3_OH) that is partially overlapped by the intense Si–O absorption band of flint itself (Fig. 6f). ﻿Indeed, all micro-FTIR spectra show a very intense band at 1157 cm^−1^ (Si–O stretching mode) and less intense bands at 469 and 798 cm^−1^ (O–Si–O, and O-Si-Al bending modes), as is characteristic of the cryptocrystalline silica (SiO_2_) which is the principal constituent of flint^75–78^. Furthermore, all peaks show an up-down reversal due to the restrahlen effect^79,80^. Despite the fact that the residue signal is masked by the stone absorption spectrum, the peak at 913 cm^−1^ is confidently assigned to the mineral constituent of bone whose attribution is furthermore supported by their elemental characterization.

On two of the remaining three tools, FTIR detected no residues, while the third specimen showed the presence of a doublet at the frequency of 1575/1536 cm^−1^ that is attributable to the C–O stretching vibrations of the carboxylate group of fatty acid salts, along with the C–H stretching vibrations of organic components detected in the range of 3000–2800 cm^−1^ (Fig. 7e). Based on our experimental reference collection of hundreds of FTIR spectra, and on the available literature, we propose their assignment to adipocere, a wax-like organic substance due to the anaerobic bacterial hydrolysis of fat tissues and mainly constituted by fatty acids such as myristic, palmitic, and stearic acids^83,84^ (see Supplementary Information S1 for more details).

Especially noteworthy is the correlation between the macro-residue detected through the optical microscope and the FTIR results obtained during the inspection of the eighth tool (retouched flake #35 AW13d 71.14-08). On the same edge where the mass of birefringent fat droplets was observed, we detected the presence of micro-residues of adipocere in association with C–H stretching vibrations of organic components as the result of contact with greasy material (Fig. 7d,e).

Of the additional 14 tools that showed no visible macro residue, we found evidence of micro-residues of bone only on the new active edge of one tool. FTIR analysis detected bone on the active edges of two tools and recorded micro-residues of adipocere on six tools (Supplementary Table S2).

As can be seen in Supplementary Table S2, micro-residues were consistently recorded along the new active edges, with the exception of three tools that exhibit, respectively, residues of bone (2 cases) and adipocere (1 case) smeared along their old active edges. However, these residues were also found on the new modified edges in close association with the use-related scars that are clearly from the last use-cycle, a finding which cannot be ignored. We assume that the residue’s location might be a result of a displacement of the organic materials worked during the second use-life of the items, and thus cannot serve as an indication of their original use (i.e., the old edge). This is even more likely in the case of the item with the adipocere micro-residues, since adipocere is soap-like in texture. As such, adipocere can easily spread all over the tool’s surface when animal materials are processed. For instance, the retouched flake reported with adipocere micro-residues (#35 AW13d 71.14-08) bears traces of adipocere on both the old and new active edges. However, since the new retouch cut the old active edge (thus, visually seen as a continuation of the old one), we associated the use-wear and residues found on the item only with the new active edge.

Aside from the detected residues attributed to animal materials and their strong correlation with the tool’s function, we also found micro-residues that are not related to their ancient use. For example, we found calcite accumulations on a small number of items (Fig. 4d), and we were able to distinguish them from bone micro-residues because of their unique topographical features (clearly detectable under the scanning electron microscope despite their close resemblance to bone residue when compared through only the lens of optical light microscopes) and their calcium carbonate composition.

A bright white accumulation, composed of high percentage of barium, was also found along the new active edge of one of the items. Barium is a metallic alkaline earth metal that is probably a natural element in the sediment at Revadim. Thus, the presence of barium on the item is probably related to the sediment in which the item was buried and from which it was unearthed during excavation.

Finally, modern contamination in the form of microscopic human skin particles (detected through SEM) and modern fibers were noticed on a small group of items during the first screening of the sample and before the cleaning procedure. Non-used related residues were photographed and documented and subsequently removed following the cleaning procedure prior the residue and use-wear analyses (see method section and Supplementary Information S1).

#### Final considerations

All in all, the identified residues show distribution patterns, morphological features, and spectroscopic characterizations similar to those of previously analyzed lithic materials from Revadim C3^47,71,85,86^ (Supplementary Table S2).

Bone and adipocere are the two recurrent types of residues found on the fully patinated PPF tools, as well as on other analyzed tools from layer C3^47,71^. This is due to the favorable taphonomic conditions at this location after the abandonment of the tools^47^. A combination of different factors, including the presence of carbonate-rich solutions and heavy metals (e.g., barium, manganese, and iron), created an alkaline anoxic environment where microbial activity was reduced, thus allowing the preservation of the two most long-lasting animal residues: hydroxyapatite and adipocere. Bone (namely its mineral part) is well preserved in alkaline soil with pH above 5.3^82,87^, while adipocere is easily formed in mildly alkaline soil^88,89^. We want to stress that neither the micro-residues of bone, nor those of adipocere, result from modern or environmental contamination (see experimental results in the Supplementary Information S1 and Supplementary Fig. S5b,c). Adipocere was formed and was able to survive thanks to the taphonomical conditions that occurred at Revadim layer C3. If the case was of modern contamination, we would have expected the presence of adipocere on a major percentage of tools (notwithstanding that the tools were always manipulated with gloves and analyzed after cleaning, according to our methodological protocol) including those in the sample that did not present any associated residues or use-wear. To the contrary, their ancient origin is confirmed by their appearance, consistency, quantity, distribution over the tool surfaces, and more important, by their correspondence with the use-wear traces.

The use-wear and residue results clearly show extensive animal processing using different tools at Revadim layer C3, in combination with other activities which surely involved the treatment of vegetal materials^90^.

## Discussion

The use of fully patinated PPF tools varied during their first life cycle. We identified longitudinal motions associated with cutting on four items from the sample, based on their use-wear results. We also detected transversal motions associated with the presence of a retouched edge on three items (identified as scraping). One item was used for mixed activities, another for chopping, while three others were possibly also used in longitudinal motions although the signs on their old edges were not sufficiently determinative. The processed materials were mostly soft to medium, and in two cases even hard.

The varying degrees of patina on the tools’ older surfaces hampered the residue analysis of the old edges of all items. Thus, no worked materials could be reconstructed from the old edges. In the case of three items, bone residues (double-checked through two different and independent analytical techniques) were found on the old edges; however, these residues are not believed to be related to the original use of the items pre-patination but rather to be a result of displacement of the organic materials worked with the new edges, during the second use-life of the items.

The better-preserved new edges in the sample provided use-wear results for the second life cycle of 25 tools: most tools (n = 22) were used in transversal motions identified as scraping, including all tools that were techno-typologically defined as scrapers based on their recycled new edges prior to the functional analysis (aside from one that was indeterminable). Two additional tools were used in a combination of transversal and longitudinal motions (scraping and cutting, respectively). The chopper in the sample, used for chopping in its first life cycle, was also used for chopping during its new life cycle. The processed materials were mostly soft-medium to medium, while in the case of five items the processed materials were medium-hard.

The well-preserved lithic micro-surfaces of the new active edges of two items—the chopping tool (#49 AQ17c71.03-70.01) and a retouched flake (#29 AV13a71.13-07)—allowed us to make a more detailed characterization of the worked materials. Both showed to have been in contact with bone. This was evident from the smooth bone-like polish with a domed topography observed on the surfaces of the new edges, used, respectively, for chopping and scraping activities. The latter probably occurred during butchery.

From the presence of animal micro-residues in the form of hydroxyapatite, fat and adipocere on 13 of the tools (detected through optical microscopes, SEM–EDX, and FTIR analyses), we can deduce that their new life cycle was dedicated to the processing of animal carcasses.

Eight tools provided use-wear results on both their old and new edges. For six of these eight items, the activities performed with the old and new edges showed no correspondence. Two exceptions were the chopping tool, which shows traces of chopping on both its old and new active edges, and another retouched flake, which shows traces of scraping on both the old and new edges. The other six tools were recycled and modified for a new, different purpose.

Many of the new modifications show a tendency toward concave shaping, with the tool subsequently used to perform scraping motions. This correlation between form and function has already been noted in the study of small, recycled flakes in Area C3, and seems to be part of the techno-functional habits of the Revadim inhabitants^46^. This is especially interesting in light of our use-wear results in this study: while the indicative first-cycle edges mainly bear evidence of cutting motions, along with some evidence of scraping, the second-cycle new active edges were used almost exclusively for scraping (Supplementary Table S1 and Fig. S8).

Moreover, the specific but minimal reshaping used to create these new edges strongly suggests that their collectors have chosen to preserve these items as they were as much as possible, while still providing them with new functional potential. The inhabitants of C-3 Revadim could easily have chosen instead to create these tools from fresh flint blanks, as scrapers made of fresh flint were indeed found in C-3 together with the discussed recycled ones.

While it is not definitively known why these ‘old’ patinated tools were collected and minimally modified, we argue that ‘old’ patinated tools were selected and reincorporated into the functional tool kits of the site’s inhabitants for their perceived technical knapping potency gained during their original, old, life cycle. Unfortunately, the needed technological properties considered while collecting ‘old’ patinated items for making new tools are unknown since standardization among the tools in the different typological categories is lacking (PPF tools and tools made of fresh flint alike). Thus, tools from all types were created on various blank types that vary in size and shape and ‘old’ patinated blanks were chosen for recycling based on the morphological needs desired at a certain moment for the creation of a certain wanted tool.

We further suggest that other considerations also played a role in the decision-making process. While some scholars will favor “least-cost, least-effort” as the most likely reason behind the collection, recycling and re-use of ‘old’ patinated items, we suggest another hypothesis. In our opinion, the notably minimal reshaping of the new edges strongly suggests that their collectors chose to preserve the original appearance of the items as much as possible, while still providing them with new functional potential. This decision was made not because of scarcity or shortage, and nor was it practiced as a shortcut procedure. Our arguments are based on the following reasoning:

- Fresh unpatinated tools are present alongside PPF tools, and constitute the majority of items found within the tool category (82%). Although PPF tools appear in significant quantities, they still constitute only 18% of the tool category, meaning that this is not the main tool-production strategy. So, we contend that a “least-cost, least-effort” explanation is unlikely in this case, as otherwise, why not execute this strategy to a larger extent as ‘old’ tools were readily available?
- Of the noteworthy number of tools in layer C3 that are made from ‘old’ items (18%), the majority (75%) preserved most of the appearance of the ‘old’ blank. While collecting suitable tools and minimally reshaping them might indicate a practical decision, the probable mnemonic significance of these old surfaces should not be ignored.
- Fresh unpatinated scrapers were found in layer C-3 alongside the sample of PPF tools discussed here, indicating a preference for using fresh flint for scraper production.
- Conceiving and creating a new tool from an existing patinated artifact (whose shape and size were preserved) is not necessarily easier than creating a tool from scratch. It differs greatly from creating a new tool from a newly produced blank and leaves the creator only a limited set of options for styling and reshaping

We contend that the collection and preservation of the patinated blanks in the process of the incorporation of ‘old’ into a new tool kit via recycling stemmed both from functional intentions as well as the desire to preserve important elements and characteristics of the collected items (i.e., the ‘old’ patinated scars and modified surfaces). We suggest that mnemonic memory^62,66,69^ plays a role here. We work under the assumption that in the process of ‘collection’, it is mnemonic memory that dictates the recognition and collection of ‘old’ human-made items. In the process of ‘modification’, it is mnemonic memory again that dictates the pattern of recycling to preserve the original surfaces and visual markers of the original collected item while adding new features to it^58,60^. We put forward the hypothesis that these items served as significant ‘memory objects’ for their collectors, for whom they played the following roles:

- To preserve past human creation. While collecting the patinated items, people must have recognized that they had been modified before. Moreover, in the process of recycling, the knapper might intentionally have chosen to preserve the previous modified surfaces and active edges of the old patinated tool out of appreciation for the work of earlier humans (ancestors?), as well as out of a sense of familiarity with the process of knapping^58,91^.
- To preserve the biographies and itineraries that are visually presented on the item itself^58,59,68,69,91^, in order to relate, remember, appreciate, and preserve the journey the item has made until the point of its collection.
- To serve as mnemonic devices for their new owners, for whom they might have evoked private memories and experiences. These private memories might have borne no relation to the old patinated item itself, which nonetheless might have conjured other places, times, individuals, events, or even just a sense of familiarity^58,62,66,69,91,92^.

In this way, the knappers and users managed to preserve and highlight the item’s itineraries and accumulated significance. This purposeful pattern might have stemmed from the inhabitants’ desire to preserve the memory of older groups (in the sense of “gifts of the ancestors”)^93^, familiar/important locations (in the sense of “pieces of places”)^94^, as well as private memories, associations, and experiences (“mnemonic memories and experiences”)^58,69^. Thus, we suggest that the preservation of memory was both visual and physical: the old item’s original appearance and patinated surfaces were preserved as much as possible, as well as their original and more recent functionality.

**Figure 8:**
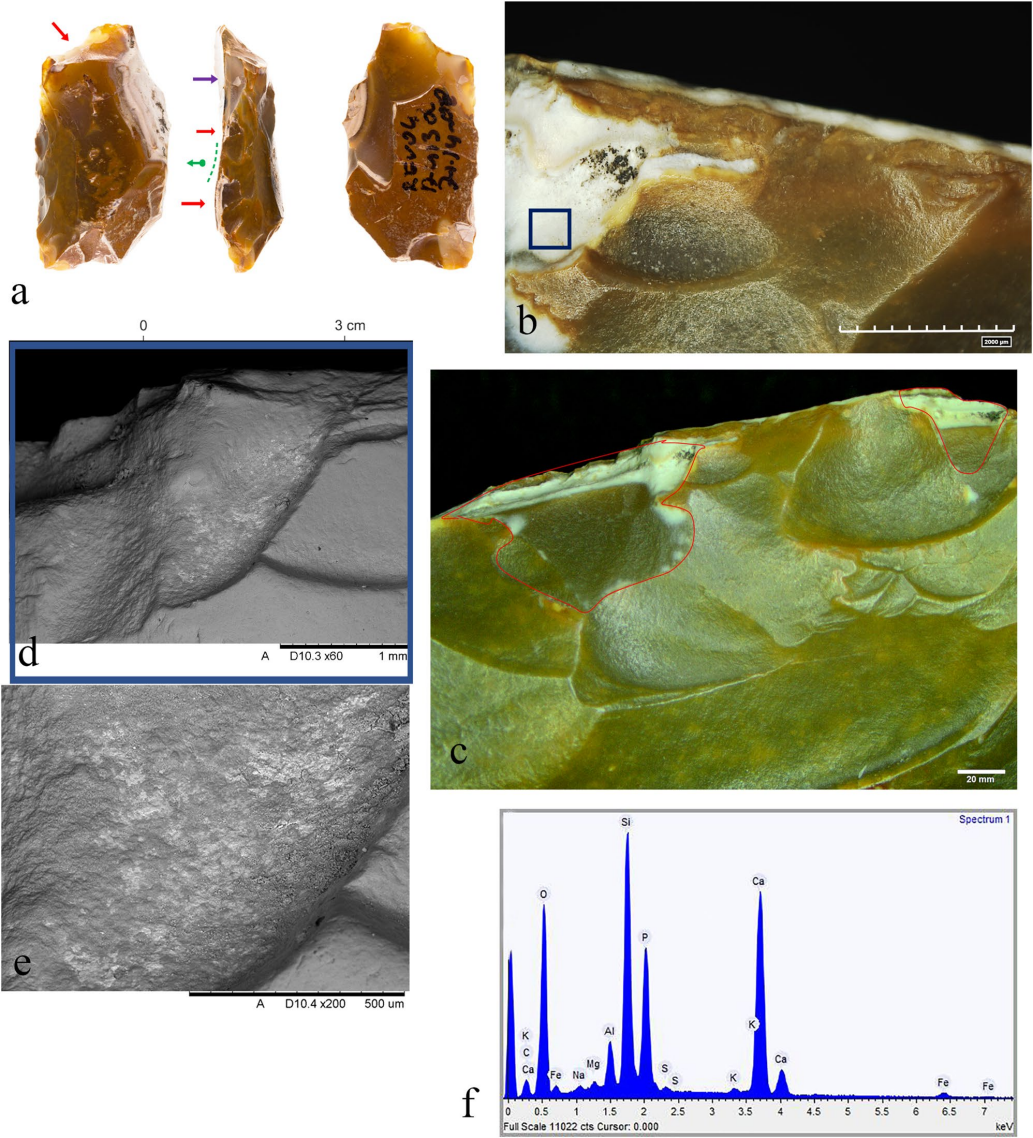
(**a**) Scraper (#6, Aw3d 71.14-08) showing a small portion of its original, old, active edge (green dotted line), and two later modifications (red arrows). The purple arrows mark the location of a modern fracture; (**b**) edge removals along the item’s old active edge; (**c**) the active edge showing old and new modifications (delineated in red). The blue square marks the location of the bone micro-residues; (**d**,**e**) SEM-micro graphs showing the new modified edges and the micro-residues of bone found on them (notice the smoothness of the surfaces due to the heavy patination in (**d**); (**f**) elemental analysis of bone micro-residues.

A noteworthy example is provided by one of the sampled side scrapers (#6 AW13d 71.14-08), which visually presents its biography and itineraries in two phases of recycling, demonstrated by three different stages of patination (Fig. 8a). The older phase is represented by a patinated flaked surface that is brownish opaque in color and covers most of the dorsal and ventral faces of the flaked item. After its first life cycle ended and the abandoned object became covered in patina, it evoked someone else’s curiosity^60,68^ or ignited a mnemonic experience^62,66,69^. The patinated flaked item was then collected and reshaped into a scraper that left intact most of the original item’s morphology and patination. Its past connotations were chosen to be preserved during its next life cycle. In this way, the item’s previous roles, uses, and meanings were kept as a memory while new roles, uses, meanings, and mnemonic values were added^58^.

The patina related to the scraper’s second life cycle is translucent and light brown in color. However, due to the high degree of rounding, it was not possible to reliably identify the consistency of the processed material (green arrow in Fig. 8a). As the second life cycle of the object came to an end (abandoned again and covered in a new layer of patina that is different in color), it evoked someone else’s curiosity and was collected again. The last and most recent life cycle of the item is represented by two removals along the older active edge (red arrow in Fig. 8a), cutting the previous edge removals that occurred during its second phase of life as a scraper (Fig. 8c). The edge removals from the scraper’s final use-cycle would be even more prominent were it not for the two scars that occurred along the item’s outer edge, exposing the typical chalk-white aspect of the desilicated flint (Fig. 8b).

Micro-residues of bone were observed and recorded embedded within one of the two scars attributed to the scraper’s final use-cycle, but in the absence of reliable wear traces we are unable to correlate their presence to a specific activity (Fig. 8d–f). However, we suggest that, in its final life cycle, this item came into contact with bone. Finally, excavation or post-excavation damage at one of the extremities of the scraper’s active edge (purple arrow in Fig. 8a) should also be noted.

The analysis of the fully patinated PPF items from layer C3 also provides some insights into the time-related pattern of human groups visiting the site of Revadim, which in turn might shed light on human-object and human-landscape relationships. It appears that late Acheulian groups repeatedly returned to localities such as Revadim, where the remains of earlier human activities were still evident, in order to select older artifacts. Such behavior is not unique to early humans, as it was also observed among other primates, and thus might be deeply rooted in human ancestry. Worthy of mention is such behavior among chimpanzees and Japanese macaques, who repeatedly visit specific locations where stones were used and/or cached while purposefully selecting and using previously used stones^95,96^. This was suggested to be an aspect of ritualized behavior among chimpanzees, including the recurring practice of selecting previously thrown stones and re-throwing these against the same tree^96^.While it is difficult to determine a clear reason behind the chimpanzee practice of throwing stones at specific trees and creating stone-cairns, a few possibilities have been suggested^96–98^.

We suggest that in the process of recycling, the late Acheulian knappers of Revadim intentionally preserved the previous modified patinated surfaces of items they collected from older localities at the site. We argue they did so out of appreciation for the work of a human ancestor who previously occupied the site, as well as out of a genuine sense of familiarity with the technological trajectories practiced in the past^93^. We should also consider the possibility that the locations from which the old patinated tools were collected were of importance to the collectors (e.g., the concept of "pieces of places")^94^. Tools collected from older localities of the Revadim site itself might have been charged with additional ontological and memory value that derives from Revadim as a multi-layered site of recurrent human occupations^94^. The history of human occupations might be visually preserved on the old tools by their colorful patinas.

## Conclusions

The exceptional preservation of the lithic items of layer C3, together with the integration of microscopic observations and elemental and chemical analysis of both use-wear and residues, allowed us to trace the life cycles and functional aspects of 28 fully patinated PPF tools from late Acheulian Revadim (57% of a sample of 49 items). The results provide additional unequivocal evidence that many of the sampled items underwent two life cycles and further support the hypothesis that these items were in fact old, shaped tools that were discarded, covered in patina, and then later collected and reshaped to function as tools again.

The fully patinated PPF tools served various purposes during their first life cycles. The ‘old’ patinated active edges mainly bear evidence of cutting, along with some examples that bear evidence of scraping, mixed activities and chopping. The tools were used on mostly soft to medium materials. During their second life cycle, most items (n = 22) were used for scraping. The chopping tool (used for chopping) and one retouched flake (used for scraping) came in contact with bone during their second life cycle, the latter probably during butchery. Residue analysis further revealed that 13 tools were used for the processing of animal carcasses. Eight tools provided use-wear results on both their new and old edges. Six of them were collected, recycled and modified for a new, different purpose: their first life cycle is characterized by cutting motions and their second by scraping motions.

This study adds another piece to the puzzle of collecting and recycling old tools. Our first comprehensive attempt to describe the multiple life cycles of PPF items using functional approach shows clear promise for the analysis of fully patinated items in the future. Furthermore, the study shows once again that the Revadim hominins were able to conceive a wide range of tools using multiple technical trajectories.

## Materials and methods

Fully patinated PPF tools from layer C3-East + West are the focus of this study. The lithic assemblage of layer C3 at Revadim comprises 28,444 items (débitage, debris, and tools). In total, 2,546 tools were identified within the lithic assemblage of layer C3 (Supplementary Table S3). Among them, PPF shaped items were identified within the tool category of Revadim layer C3 East + West in significant numbers (n. 461). Out of the 461 PPF tools, 75% (n = 345) were fully patinated.

PPF items were identified and processed following the methodology applied by Efrati et al.^34^, by Belfer-Cohen and Bar Yosef^27^, and following the definition of Goodwin^15^ for ‘double patina’: items ‘that have been modified again, thus leaving newer scars in unpatinated, or less patinated, condition’ (p. 68). When ‘old’ flaked surfaces could not be confidently distinguished from old cleavage surfaces or natural surfaces (e.g., cortex/neo-cortex), the items were not included in the PPF category. Hence, the numbers presented should be regarded as minimum estimations of the phenomenon in the researched area.

This is a preliminary analysis, and thus we opted to build a sample that is large enough for meaningful use-wear and residue analyses, but not so large as to be impractical. We were aiming mostly at assessing the potential and success rate of this type of analysis in general, and in the case of Revadim layer C3 in particular, an archaeological context that we chose for its excellent use-wear and residue analysis results in prior studies^46,47,71,86^. We ended up sampling and analyzing 49 fully patinated PPF tools from layer C3, Revadim for use-wear and residue (hereafter, ‘the sample’). The main criteria for selection were the presence and visibility of at least two potential shaped active edges: items with at least one ‘older’ active edge (covered in patina), and at least one ‘newer’ (recycled) active edge. The sample comprised 17 retouched flakes, 10 side scrapers, 10 notches, four varia tools, three chopping tools, three denticulates, and two retouched special spalls (items removed from tools during resharpening).

### Use-wear analysis

We conducted a preliminary screening of the sample at the Laboratory of Prehistoric Archaeology at Tel Aviv University, to assess its state of preservation, presence/absence of ancient or modern residues, and to investigate the potential of old and new active edges. We screened the specimens using a Zeiss Discovery stereomicroscope (zoom up to 8 ×, objective 1 ×, oculars 10 ×, and equipped with a LED ring-light) and a Zeiss Axio Scope A1 (magnification ranging from 50 × to 500 ×).

The sample was then sent to the Laboratory of Technological and Functional Analyses of Prehistoric Artifacts at Sapienza University of Rome (LTFAPA), where we carried out the complete functional and residue analyses. The use-wear analysis combined observations at low and high magnifications^99–102^, using a Nikon SM stereomicroscope with a 1 × objective, a 10 × ocular, and a 0.75 × e7.5 × magnification zoom in reflected light. We documented microscopic wear marks with a Nikon Elite metallographic microscope in reflected light with a 10 × ocular and 10 ×, 20 × and 50 × objectives equipped with a reflected differential interference contrast (DIC). A Hirox RH-2000 digital microscope covering a magnification range of 35 ×–2500 × was also used for both edge removals and micro wear inspection.

We compared edge removals and micro polish with the extensive experimental use-wear comparative collection stored at the LTFAPA, as well as with experimental flakes and tools produced and used in the framework of previous functional studies on Revadim assemblages^46,47,71^.

### Residue analysis

We observed archeological residues before and after the cleaning procedures. First, we inspected them with the Hirox RH-2000 digital microscope and morphologically described them according to their appearance, consistency, color, inclusions, birefringence, and spatial pattern of distribution. Once documented, they were subjected to FTIR and SEM–EDX analysis.

Micro-FTIR was applied using a Bruker Optic Alpha-R portable interferometer with an external reflectance head covering a circular area of about 5 mm in diameter. We placed the samples directly in front of the objective, without preliminary treatments, and different spots for analysis were selected. The investigated spectral range was equal to 7000–375 cm^−1^, with a resolution of 4 cm^−1^, and at least 250 scans were performed. Infra-red measures were taken from at least two points on each active edge (old and new) that showed use-related scars on both dorsal and ventral surfaces, and from at least two points on the inner dorsal and ventral surfaces of each item.

We performed electron dispersive X-ray (EDX) analysis with a Hitachi TM3000 scanning electron microscope (SEM), equipped with a SwiftED3000 energy dispersive X-ray spectrophotometer. We used different accelerating voltages during the analysis of residues: 5 kV was used to characterize topographic and textural traits, while 15 kV mode provided elemental information through grey-scale images according to the atomic number using the high sensitivity backscattered electron detector. We performed the electron dispersive X-ray spectroscopy on each identified residue. Two to three measurements, from different locations, were taken from each residue using 15 kV accelerating voltage in BSE mode with a magnification of 500 × to 800 × and an acquisition time of 400 s.

We took control samples on two points of tool surface for both FTIR and EDX analyses. This allowed us to check the distribution of residues and distinguish between use-related and post-depositional residues. The interpretation of residues was supported by a large FTIR and EDX spectra reference collection of experimental micro-residues on stone tools available at the LTFAPA and assisted by previous studies on Levantine Middle Pleistocene assemblages^46,47,71,103,104^ (see Supplementary Information S1). Additional comparisons were also made with the available literature^73,74,76,77,81,105–109^.

### Cleaning protocol

We washed all items two to three times. First, under tap water, and later in a bath of demineralized water in an ultrasonic tank for 10 min. The specimens that underwent the elemental and chemical analyses were then washed again in demineralized water in an ultrasonic tank for 5 min prior to the analysis.

We used powder-free sterile gloves each time we came in contact with the items throughout the entire process of analysis, following the methodological protocol for use-wear and residue analyses; during the identification process of PPF items in the tools category of Layer C3, during the creation of the sample for the study, during the cleaning process, and during the analysis procedures themselves. We also used Parafilm (laboratory film) to wrap the modeling clay supporting the pieces during the observations at optical light microscopes.

### Theoretical background

Anthropological and archaeological theories claiming a shared relational frame of interaction between human and non-human agents have become prevalent in recent years, mostly following the ontological turn^110–113^. The ontological turn offers fresh perspectives on personhood, sociality, and human-non-human relationships^112,114,115^, based on the recognition and acceptance of the different ontologies at work in the world. In this theoretical framework, metaphysical theories^59,68,113,115–129^, together with ethnographic studies from various environmental settings^120,123,130–139^, are applied in order to better understand the practices of present and past hunter-gatherer groups. Ethnographic and archaeological literature shows that present and past indigenous societies are constantly engaged in establishing and maintaining social relations between themselves and other-than-human persons^120,130,134,137,140–142^, all to ensure that current ways of life will be sustained^138,142,143^.

Under this view, humans are active agents among many others; thus, they are expected to live together with other persons (human and non-human), maintain good relationships with them, and respect them to maintain world orders and achieve well-being^112,113,136,138,140,144–146^. This worldview is given vital expression in the duality of humans perceiving animals, natural resources, and objects as other-than-human persons and equal agents of a shared habitat while also still exploiting and using them (e.g., hunting animals and consuming natural resources)^136,138,142,146,147^.

Ethnographic examples show that in numerous non-Western cultural contexts, dichotomies (e.g., human-non-human, mind–body, culture-nature) are not as we understand and live them today^143,148^. Indigenous groups worldwide live by the assumption that animals, plants, natural resources, and material objects are equal active agents^120,123,130,134,136–141,149^. Several studies even explore the possibility that modern Western dichotomies did not exist during prehistoric times. For example, a study that attempted to identify and analyze similar dichotomies and how they may have been understood in Early Neolithic Europe (ca. 5300–4900 cal. BC) shows that the fixity of categories that we are familiar with today did not apply back then. Dichotomies such as culture-nature, farmer-hunter, and human-non-human may not have been recognized as such by Neolithic people in Central Europe^150^. A similar conclusion was reached on the persistence of nature-culture dichotomies in the field of body studies^148^, dichotomies that may not have applied during prehistoric times.

Lifeways vary, of course, from one group to another due to different ecological adaptations and cultural values^151,152^. However, the universality of some ‘core values’ in the ‘mode of thought’^153^ of most indigenous groups can still be argued for, given the similarities in their expression (e.g., sharing, self-provisioning, personal autonomy, egalitarianism, and animism)^137,146,154–156^. We contend that such universal values, shared by contemporary indigenous groups, were also shared and practiced by past hunter-gatherer groups to some extent, even during the Lower Paleolithic. Reciprocal relationships with other-than-human persons that share the same environment^143,146,157,158^ are reflected in activities both sacred and mundane. We know that prehistoric groups, from the Lower Paleolithic onwards, collected items from their surroundings or chose materials for the production of tools not only for their functional properties but for their aesthetic ones as well (e.g., color and shape)^34,57,58,93,159^. The extraction and use of bird feathers and talons^160–165^ exemplifies this idea as well. Neanderthals, during European Middle Paleolithic times, are known to have entered deep, dark caves to build circular structures and used fire while in the cave^166^, climbed up a volcano soon after it erupted^167^, and dived into deep water in order to retrieve fresh shells for making tools^168^. In our view, all these behaviors might be viewed as practices rooted in ontological beliefs.

#### The role and place of objects

Human relationships with non-human objects have become an essential theoretical component in sociology, anthropology, and archaeology^59,117–119,126–128,169,170^. The view that objects are imbued with agency and should be seen as ‘object-persons’ affords us the possibility to reassess previously ignored or overlooked aspects of material culture and reexamine the significance of daily and non-daily features that these objects might represent and encompass^125,143^. In contemporary indigenous societies, for example, daily objects and the materials from which they are made (e.g., stones and stone tools) are not perceived as solely passive objects intended for economic and functional use^67^. On the contrary, objects and materials are agents through which humans maintain relations, while also serving as economic and functional mediums.

Likewise, in archaeology, the production and use of tools can be viewed not only as functional but also as another way to maintain relations with the elements of the world. These items reflect perceptions transmitted over generations regarding the relationships between humans and other-than-human persons^57,123,139,143,170^. Under the frameworks of the ontological turn, tools thus constitute the means of interaction with the elements of the world and the means to form and maintain relationships with these elements^58,140,143,170–172^. Moreover, for past hunter-gatherer groups, the very process of making a tool—selecting the material from which the tool will be made, for example, or the formation of its shape—was likely seen as a means to maintain these relationships. Ethnographic and archaeological studies further suggest that tools represent, in addition to their functionality, symbolic conventions of modern and past human societies. These symbolic features are reflected in the choice of materials. They are reflected, too, in qualities such as texture, shimmer, color, and polish^65,67,158,159,173–179^ or in the location where these materials are found^58,94,179^. We also suggest that the biography of these items—what they have ‘been through’—is another vital component that adds to their symbolic meaning^58,93,139,179^. Several studies also discuss their essential roles as facilitators of social and cultural engagements^93,139,140,158,172^.

## Supplementary Information

Supplementary Information.
