# Supplementary material for: Function, life histories, and biographies of Lower Paleolithic patinated flint tools from Late Acheulian Revadim, Israel
Source: Sci Rep. 2022 Mar 3;12:2885. doi: 10.1038/s41598-022-06823-2 (PMC8894402; doi:10.1038/s41598-022-06823-2)
Supplement: Supplementary file 1 — Supplementary Information. [file 41598_2022_6823_MOESM1_ESM.docx]

# **Function, life histories, and biographies of Lower Paleolithic patinated flint tools from Late Acheulian Revadim, Israel**

**Supplementary Information**

**Bar Efrati^1*^, Ran Barkai^1^, Stella Nunziante Cesaro^2^ & Flavia Venditti^3,4*^**

^1^ Department of Archaeology and Ancient Near East Cultures, Tel Aviv University, POB 39040, Tel Aviv, 66978, Israel.

^2^ Scientific Methodologies Applied to Cultural Heritage (SMATCH), Rome, Italy.

^3^ Department of Early Prehistory and Quaternary Ecology, University of Tübingen, Schloß Hohentübingen, Burgsteige 11, 72070, Tübingen, Germany.

^4^ LTFAPA Laboratory, Department of Classics, Sapienza University of Rome, P.le Aldo Moro, Rome, 00185, Italy.

^*^Corresponding authors.

*E-mail addresses:* [barefrati@mail.tau.ac.il](mailto:barefrati@mail.tau.ac.il) (B. Efrati), [flavia.venditti@uni-tuebingen.de](mailto:flavia.venditti@uni-tuebingen.de) (F. Venditti).

# **Revadim: regional settings and material**

Revadim is located on a hillock (71-73 m above sea level) on the southern coastal plain of Israel, 40 km southeast of Tel Aviv (Fig. S1a). Several excavation seasons took place at the site between 1996 and 2004 (directed by O. Marder and I. Milevski, on behalf of the Israel Antiquities Authority and the Hebrew University of Jerusalem), during which four areas (A-D) were exposed and excavated, along with several additional trenches^48-49,51,181^. The geological sequence of Revadim, dated by palaeomagnetic analysis, exhibits a normal polarity, thus suggesting an age younger than 780 kya^49^. U-Th was used to date the carbonate coating present on the lithic material unearthed at the site. The dates, ranging from 500 to 300 kyr, provided a minimum age for human occupation at the site^48,50^.

Both the lithic and the faunal assemblages unearthed at Revadim suggest that the site belongs to the Late Acheulian of the Southern Levant^49-50^. The lithic assemblages are dominated by flakes and flake tools, and are comprised of bifaces, choppers, scrapers, flakes, and flake cores, along with many recycled items (cores-on-flakes) and recycling products (small sharp flakes with two ventral faces)^32,46-47,74,85,182^. The faunal assemblages include thousands of animal bones. Analysis was performed mainly on the faunal assemblages of Area B and Area C East, with a focus on the elephant bones (*Palaeoloxodon antiquus*)^51-51,85^. In addition to elephant remains, aurochs (*Bos primigenius*), fallow deer (*Dama cf. mesopotamica*), and red deer (*Cervus elaphus*) are the most dominant species found at the site, though many additional species were also found^51^.

Use-wear and residue analyses conducted on lithic items from Revadim have already provided outstanding results, aiding in reconstructing some of the activities performed at the site. Use-wear traces coupled with residues of fat, bone, and collagen fibers were found on many small flakes produced by means of lithic recycling, which testify to their use in precision cutting of animal body parts in Area C (layer C3)^46-47^. These results provide some of the earliest direct evidence for meat processing and consumption by early humans in the Levant. Evidence for cutting and scraping activities, most probably performed during butchery, has also been found on a sample of small flakes from Area C (layer C3) that were produced by a different technological trajectory^86^. Additionally, fat residues and use-wear traces were also identified on a biface and a scraper that were found near the remains of a butchered elephant at Area B^85^. Another study, conducted on 176 lithic items from Area B and C, showed that flakes and tools were used mostly to process soft animal materials, but also for wood and vegetal materials^90^. A recent study of chopping tools from Area C layer C3 found bone residues on them. Use-wear analysis revealed that most of the chopping tools were used in pounding motions, most probably for bone breaking and marrow extraction. Some of the choppers were also used for cutting and scraping^71^.


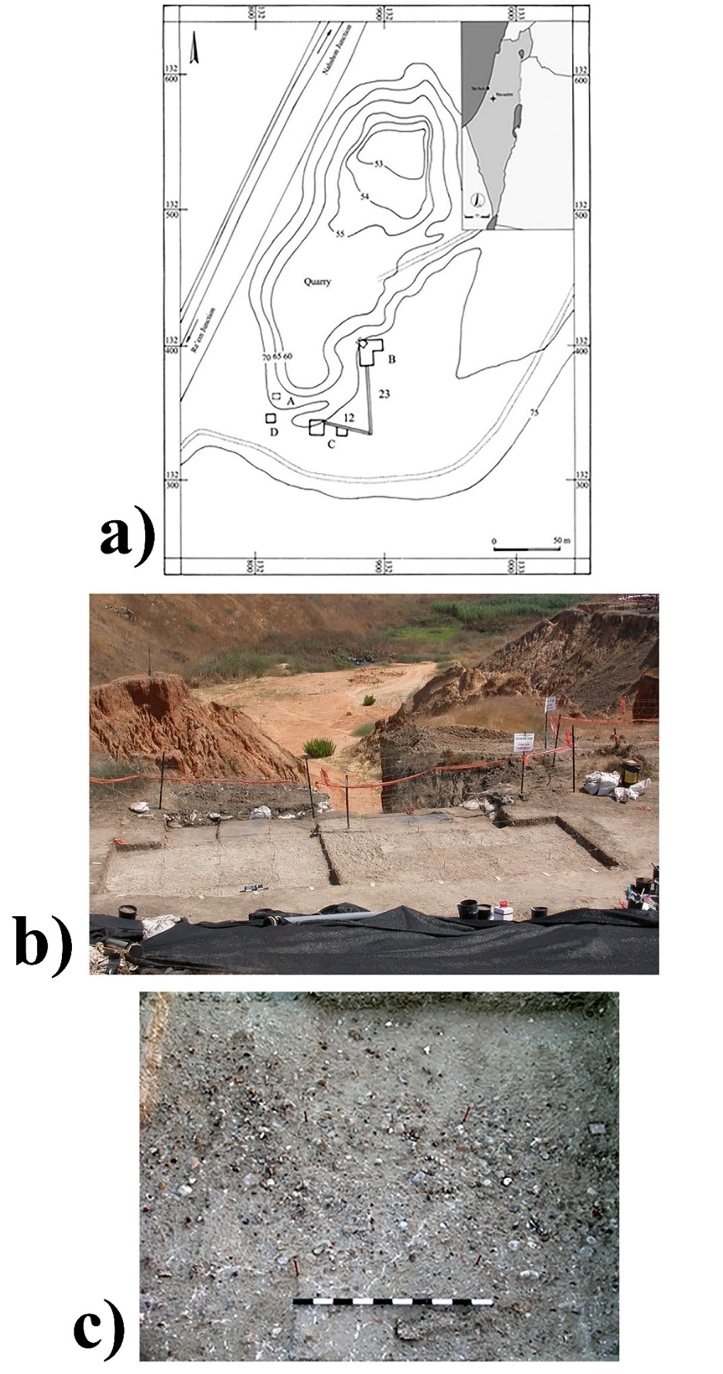


**Fig. S1.** The geographical and archaeological settings of Revadim. a) Site location and excavation areas; b) Area C, a view to the northeast; e) A close-up of layer C3.

## *Area C (layer C3)*

Area C is situated in the southern part of the site and has been divided into two separate sub-areas (C-East and C-West) over a total area of 44m^2 183^ (Fig. S1b). Area C represents the most complete stratigraphic sequence of Revadim, where five superimposed archaeological layers were discerned in sub-area C-West, which covers an area of 33m^2^ (C1-C5, from top to bottom). Area C-East covers an area of 11 m^2^ and comprises only a single layer (ca. 40 cm thick), interpreted as the continuation of layer C3 in sub-area C-West^49-50^.

Layer C3 is one of the main occupation layers in this sequence (20-40 cm thick). It contains the highest density of flint artifacts and bone in Area C^49,182^ (Fig. S1c), and is probably the result of several discrete occupation events accumulated over time into a palimpsest^47^. While layer C3 is similar to layer C2 of sub-area C-west in terms of their sedimentary and chemical composition, the two are separated by a layer of sterile sediments. The layer was divided further into two sub-layers (C3a and C3b), considered to be two distinct archaeological horizons within layer C3. In C3a, more manganese oxide nodules were observed than in C3b, where carbonate nodules were observed but manganese oxide nodules occur more sporadically^49^.

As one of the densest occupation layers of the site, from which well-preserved lithic finds were excavated, layer C3 was the focus of previous techno-typological and functional studies, all of which have produced outstanding results^32,45-47,71,86^. The current study will focus on a sample of lithic items retrieved from layer C3-East + West.

## *The phenomenon of PPF items at Revadim*

PPF shaped items were identified within the tool category of Revadim layer C3 East + West in significant numbers (n = 461, 18% of the items in this category). The patina found on the ‘old’ items varies in type, color, and texture, and differs in color and texture from the natural color of the flint (Fig. S2). The rest of the tools (82%) are made from fresh, unpatinated flint, and were found in the same context. Moreover, their relatively high frequency in the overall tool category indicates a recurrent phenomenon, and the availability of fresh flint in the close vicinity of the site also shows that lithic material was not in short supply as is the case in other sites as well^26,53,184-185^. Due to that, we believe these items were selected intentionally.

Following the preliminary work on PPF items from the terminal Lower Paleolithic site of Qesem Cave^34^, ‘old’ flaked patinated items were assumed to have been collected from the surroundings (from older sites in different environments and localities) and brought to the site to be recycled and used again. However, in the case of Revadim, as a multi-layered open-air site that was inhabited recurrently throughout the Late Acheulian, it is more likely that patinated flaked items were collected from older localities within the site itself, and then brought to the context designated by the excavators as layer C3.


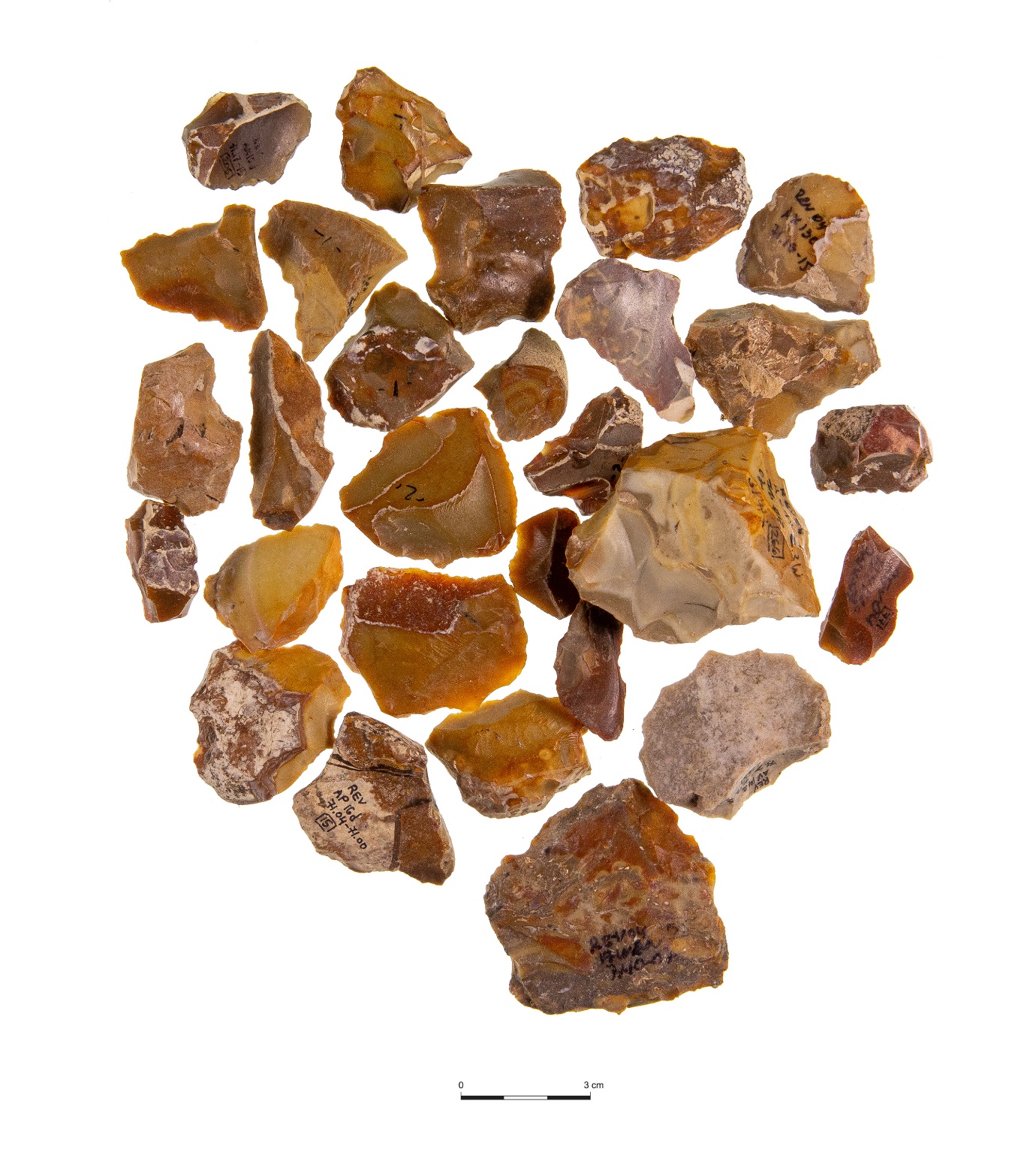


**Fig. S2.** Post-patination flaked modified items (PPF tools) from Revadim, layer C3.

The study of PPF items from Qesem Cave also suggests that ‘old’ patinated flaked items were selected for their knapping potential in relation to desired technological trajectories practiced at the site as well as their probable significance as mnemonic/biographic items. As such, they reflect, again, an educated and thoughtful choice^34,58^. The same insights appear relevant to the PPF items at Revadim. The needed technological properties that were considered while collecting ‘old’ patinated items for the making of new tools are unknown. Furthermore, the techno-typological analysis conducted on the tool assemblage of Revadim C3 (double patinated and fresh) suggests that no technological standardization exists among items associated with the same typological category, which means that tools from all types were created on a variety of blank types that highly differs in sizes and shapes. However, we still believe that a knapping potential was still observed in the ‘old’ patinated items that were chosen and collected, as a significant number of them (18%) were recycled and served as blanks for the making of new tools, even though fresh flint is available and was used on most occasions. Moreover, many PPF items from Revadim preserve most of their patinated surfaces and scar patterns. That manner of recycling preserved the morphology of the ‘old’ item. This is the case of 75% items (n=345) out of the 18% PPF tools. The new modifications on those 345 items seem to have been kept minimal and were mostly limited to shaping a new working edge. This fact, again, indicate that their morphology was needed for the desired end-product.

The preliminary analysis of PPF items from Revadim revealed two main groups. The first group consists of PPF items that were removed from ‘old’ patinated modified blanks/cores, thus exhibiting modified patinated surfaces only on their dorsal face or striking platforms. Their ventral face was detached, thus revealing the fresh color of the flint, and counts as a new modification (Fig. 1a in text).

The second group consists of complete patinated flaked items that serve as blanks for the new PPF item. These PPF items usually preserve and exhibit most of the patinated surfaces of the ‘old’ items, with few, but specific, new modifications that are usually aimed at creating/resharpening a working edge. They thus preserve most of the morphological and visual proprieties of the collected ‘old’ patinated item (Fig. 1b in text). Items of this type are of interest to us here, and they appear at Revadim in significant quantities (e.g., comprising 75% of the PPF tools in layer C3-East + West). These items, with their almost unchanged, untouched appearance, directly and visually demonstrate that they were probably collected for their preferred properties, and for their visible itineraries and mnemonic value.

# **Use-wear and residues methodology**

We analyzed the specimens through the adoption of the low and high power approaches^100-102^ for both use-wear and residue characterization. The first life/use-cycle of the tools was more difficult to reconstruct than the second due to the patination degree (Fig. S3). Their original function, when it could be determined at all, relied only on the identification of edge removals. This was informative for the detection of the motions performed and the general hardness of the materials worked. Edge removals represented by scars formed along the active edge during use were defined based on the morphology of their initiation and termination along with their distribution and orientation^70^. Their post-recycling use, however, was easier to reconstruct, also based on the edge removals observations^70^, and in some cases, on micro polish characterization. ﻿Polishes were described and interpreted based on their texture and topography along with their orientation and distribution.


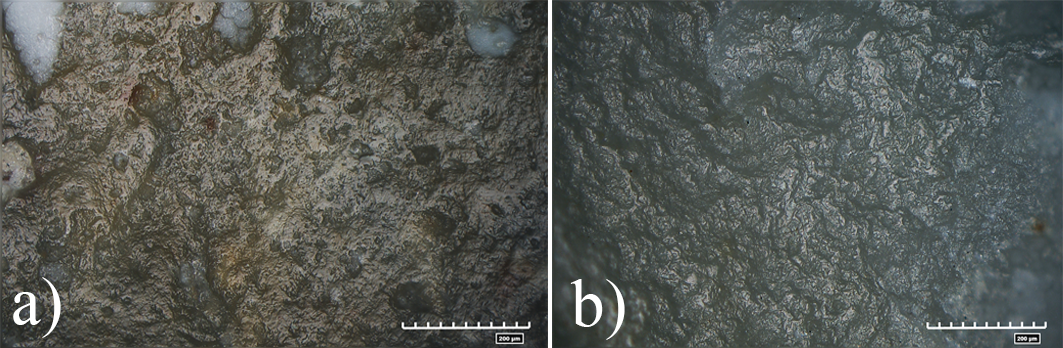


**Fig. S3.** Patination differences between the item’s old surface (a) and the item’s new surface that was exposed by the latest modification (b).

All excavated artefact from Revadim were manually handled during excavation, preliminarily sorted, and kept in plastic bags. After a limited manipulation following the excavation for purposes of a preliminary techno-typological analysis, the material was sorted and stored into separate plastic bags at the Prehistoric Archaeology Laboratory at the Tel Aviv Institute of Archaeology, Tel Aviv University. For the purpose of this study, powder-free sterile gloves were used each time we came in contact with the items throughout the entire identification and sorting process of PPF items in the tools category of Layer C3, as well as during the cleaning process, and the use-wear and residue analyses (following the methodological protocol for use-wear and residue analyses).

# **The experimental reference collection**

In addition to the published literature, we compared the use-wear and residues results on the archeological materials with the extended reference collection of experimental wears and micro-residues on stone tools available at the LTFAPA at University of Rome. Moreover, data from experimental trials performed by the same research team in the framework of previous studies on Levantine Middle Pleistocene assemblages were also used^46-47,71,103-104^. Here, we provide a summary of the experimental results useful for comparing the archeological data discussed.

## *Use-wear traces related to butchering activities*

Butchering activities include defleshing, filleting, skinning, disarticulating, dismembering, periosteum removing and marrow exctracting. These activities generate ﻿edge damage and microwear on stone tools with features proper to both soft/medium and medium to hard materials.

﻿Soft to medium materials (usually represented by meat, fat, hide, and connective tissues) are associated with feather, half-moon, and step scar terminations, as well as with snap fracturing (which may include additional smaller feather removals inside). Edge rounding is displayed when thick animal skin had to be removed from the carcass, and its rounding degree is associated with the thickness and angle of the active edge used to perform the activity. Micro polish is characterized by a bright appearance with a rough to smooth texture and a granular towards domed topography (Fig. S4a). The freshness of the worked materials are reflected in the greasy and bright polish appearance.

﻿Bone is being frequently hit throughout the butchery activity; accidentally during disarticulation, or intentionally when bones are smashed up for acquiring the marrow or being scraped while removing the periosteum. Contact with bone results in hinge and/or step scars with a discontinuous distribution along the used edge. At a microscopic level, an easily recognizable bone-polish with a smooth texture and a domed to flat topography will be apparent (Fig. S4b). When the contact with the bone is more accidental, the spotted polish along the active edge will appear more distributed. Scraping off the periosteum from the bone’s surface using a stone tool will result in a well-developed and quite observable, edge rounding; as a result of the repetitive and prolonged contact with the hard material. When bones are processed to remove the periosteum, the presence of both soft materials (e.g., the remnants of fresh fat and meat on the bone’s surface), along with the hard bone structures and its connective tissues, will develop traces associated with both classes of material. In this case, spots of bone polish will appear together with bright greasy polish along the working edge.

When stone tools such as chopping tools are used to crush bones, crushing of the pointed edge areas, compressions, and rounding of the edge will be clearly visible. At high magnification, the compressions appear as overlapped deep scars with a stepped termination and rounded outlines. Their distribution along the edge is localized around the impact point, which is usually the central area of the outer edge. Here, two types of polish are recognizable: smooth domed bone microwear polish and smooth flat bone microwear with dense parallel striations perpendicularly oriented to the edge. The orientation of the striations and features of the wear traces are consistent with chopping bones. Polish distribution is not continuous along the edge but can be observed on the points of greatest impact.

## *Residues related to butchering activities*

The heterogeneous nature of residues produced while using experimental tools in butchering reflect the different parts of the animal the tools are in contact with during the process, as well as the type of actions and gestures performed with the tools themselves (cutting, scraping, slicing, smashing etc.). The nature and distribution of the residues is also informative of the phase of the butchery process the tools take part in (skinning, disarticulation, periosteum removal, etc.), due to the occurrence and combination of specific animal residues on the tool surfaces.

Fat and bone micro residues exhibited on the archeological post-patinated flaked items are suggestive of activities involving more or less prolonged contact with bone and fat, such as dismembering, disarticulation, periosteum removing or bone crushing.

Fat and soft collagen-rich fibers are abundantly noticed during the skinning phase and disarticulation phase during our butchery experiments. Fat distribution along the edge was invasive and often formed a glossy-like film (Fig S4c). Patches of more compacted fat occurred along the outer edge in strict correlation with edge removals (Fig. S4d). Fat droplets with a specific half-moon or circular distribution have also been identified at high magnification (Fig. S4e). When used in disarticulation activities, tools trapped blood and grease and, to a lesser extent, meat, and ligament parts. When the contact with bony soft tissues is more intense, for example in the case of disarticulation or removal of fresh periosteum membrane, soft collagen fibers are mixed with fat and clustered in bands along the edge or in patches towards the prehensile areas. ﻿It is important to note that the accidental contact with bone during the butchery often results in the presence of bony fibers trapped in the edge scars together with grease and meat.

Significant patches of bone residues resulted from bone smashing, performed in our experimentation with chopping tools. When bone was being processed without periosteum, we observed wide areas below the active edge that were covered by a dense, spread mass of bony powder along with clustered masses of bony tissues appearing translucent and whitish in color (Fig. S4g-h).


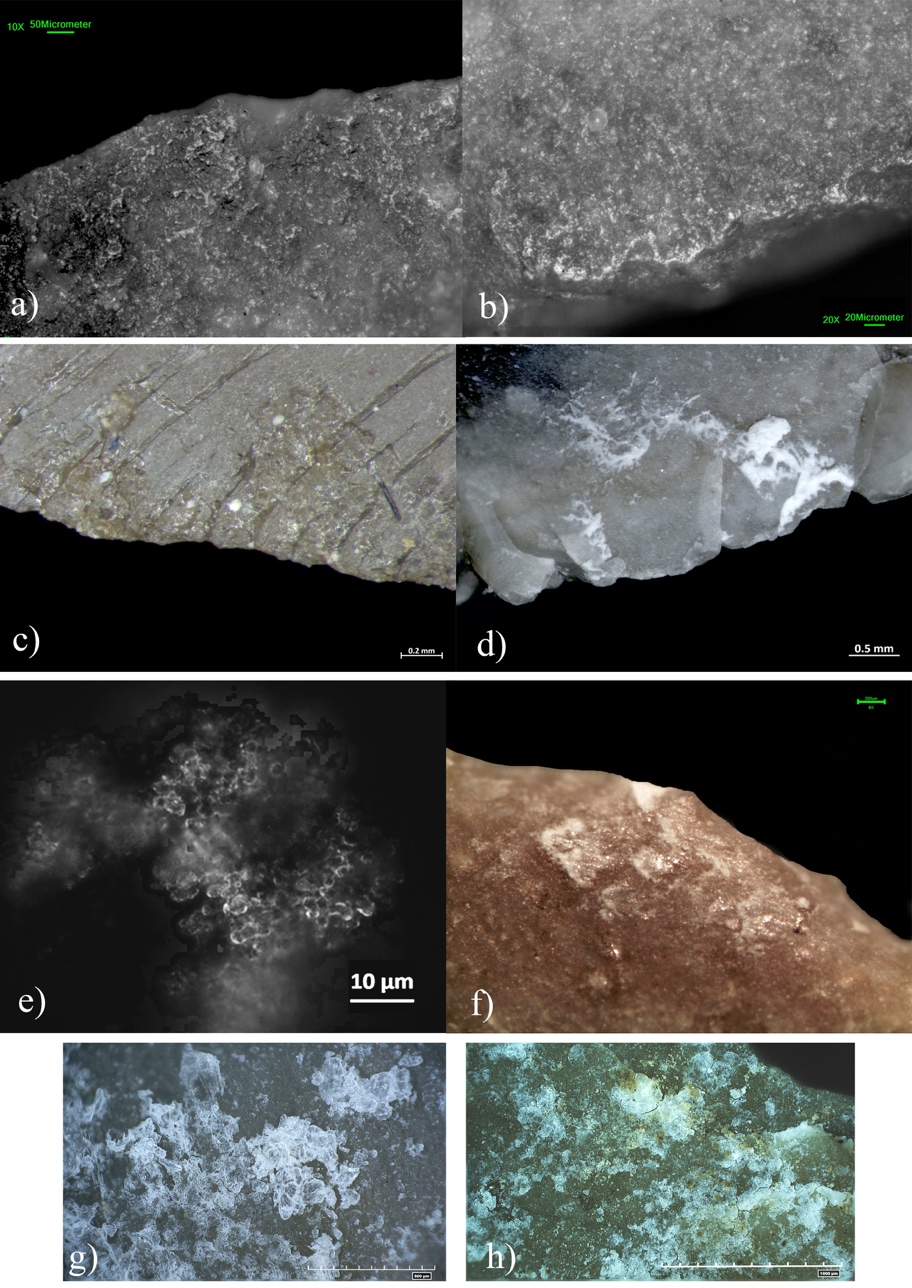


**Fig. S4.** Use-wear and use-related residues found on stone tools replica after butchery experiments. a) polish developed along the active edge of a tool used for processing metapodials; b) polish developed along the active edge of a tool used to scrape bone; c) fresh hide and fat residues on the active edge used for skinning; d) patches of fat accumulation on a tool used for disarticulate animal ribs; e) circular accumulation of superimposed birefringence fat droplets along the item’s active edge; f) bone tissues accumulation along the active edge on a tool used to process bone; g) large bone tissues accumulation after smashing fresh bone (note in h) the whitish bony tissues turned yellowish after the tool has been buried for 6 months.

## *FTIR and EDX experimental results*

The micro-FTIR spectra and SEM-EDX analysis of the replicas were analyzed under the same experimental conditions as the archaeological artefacts. We will discuss here the micro-FTIR and SEM-EDX experimental data related to bone and adipocere micro residues, and our comments once compared with the archaeological sample.

*Adipocere* is a soap-like product containing salts of mentioned acids, not soluble in water and able to invisibly fill the micro holes of the microcrystalline of flint stone surviving for centuries. ﻿It is formed as a result of bacterial activity converting fats into a mixture of fatty acids, and/or in their calcium salts. In our large database of hundreds of FTIR spectra built over 20 years of FTIR analysis performed on archeological and experimental materials, adipocere was experimentally detected only on a few items. This is because specific environmental conditions have to occur so that adipocere can form (i.e., humidity, temperature, and lack of oxygen).

For the sake of comparison, we show micro-FTIR spectra of an experimental artefact used to process hide measured on two different moments: soon after the experiment, and after 4 months (red and blue spectra in Fig. S5). While fresh fatty substances show the absorption peaks at 1636 cm^-1^ and 1530 cm^-1^ respectively assigned to C = O stretching (amide I), N-H bending and C-N stretching (amide II), the organic part is almost spectroscopically undetectable anymore after four months. However, the doublet at ﻿1575 and 1536 cm^-1^ prove the ﻿ ﻿transformation of the fresh fat tissues into adipocere.

We thus assigned the doublet at ~1573/1537 cm^-1^ to adipocere, whose formation is due to hydrolysis and hydrogenation of fatty tissues into a mixture of predominantly fatty acids as myristic, palmitic and stearic acids. Their relative amount depends on the nature of the soil. It must be said that the doublet is present in the spectra of the mentioned acid and their salts with other peaks (i.e., around 1700-1600 cm^-1^, and in the 1500 – 1400 cm^-1^ interval^186-187^, whose intensity decreases until disappearance when the amount of salts increases, leaving the doublet as the most intense IR spectroscopic peak^188^).

Based on our experience and our comparative work between the archeological and experimental results, FTIR spectra from archeological tools are not always completely comparable with FTIR spectra made on modern and fresh residues soon after the experiments, or on FTIR performed on pure matters. What we observed is that FTIR measurements performed on archeological residues show always weak bands if compared to the experimental ones (this is due to preservation condition and quantity of preserved residues). However, the spectroscopic bands discussed here are the most characteristic for bone and adipocere residues and can be interpreted as a fingerprint for their presence.


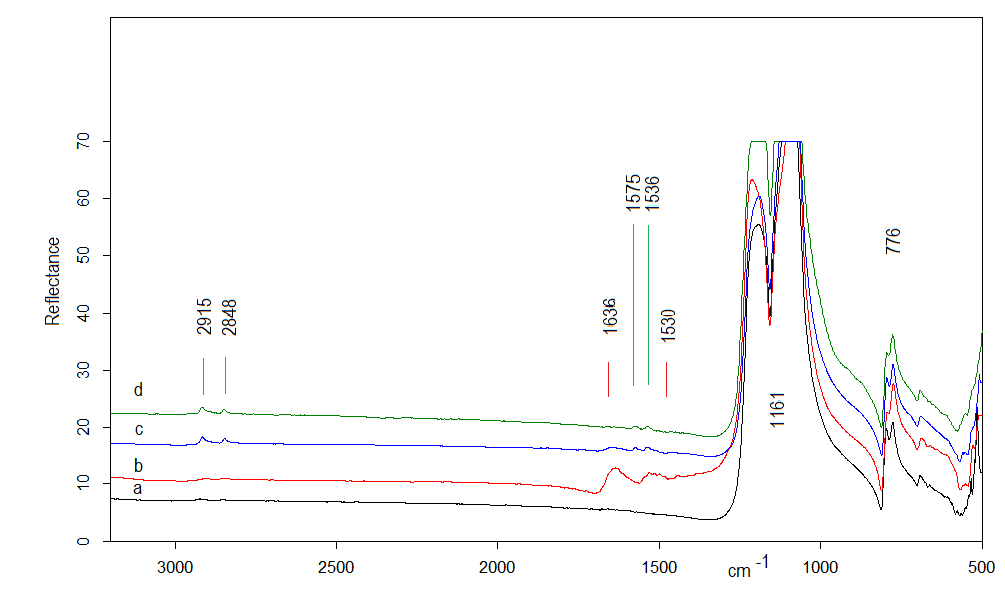


**Fig. S5.** Comparison of micro-FTIR analysis of adipocere residues. (a) Sample control micro-FTIR performed on the archeological fully PFF tool AV13a 71.18-14 showing no residues; (b) Micro-FTIR of a lump of fat measured soon after the experiment on an experimental tool used to process hide; (c) Micro-FTIR of the same experimental item performed four months after the experiment was held; (d) Micro-FTIR performed on the archeological fully PFF tool AV13a 71.18-14 showing micro residues of adipocere.

*Bone* is a resilient organic material made of the inorganic mineral hydroxyapatite [Ca5(PO4)3(OH)] ﻿which includes calcium phosphate, calcium carbonate, calcium fluoride, and calcium hydroxide. The major minerals in bone tissues are calcium and phosphorous, and ﻿the most characteristic elemental signature of bone is the calcium-phosphorus (Ca/P) ratio. Stoichiometric hydroxyapatite has been reported with a Ca/P ratio of 2.15 while modern powdered cortical bone has a mean of 1.88, with a range of 1.61–2.02^189^. The variation in the Ca/P ratio also depends on the bone types and the animal species analyzed^190^.

According to our EDX measurement of modern and ancient bone tissues, this ratio is closer to 2:1. The evaluation of this ratio is established while evaluating the percentage in weight of the two elements. For the sake of comparison, we present here the percentage in weight of the elements identified in measurements taken from three different spots of a modern fragment of a pig bone mounted on a carbon stab. As shown in Fig S6a, the phosphorous appears around the half of the calcium as percentage in weight with an average ratio equal to 1.8. We always obtained this proportion or slightly closer to 2:1 in all the EDX performed on the archeological and experimental items, albeit percentage in weight of elements may vary according to preservation condition, thickness, and size of the measured residue. Moreover, it is important to bear in mind that the Ca/P ratio also depends on technical characteristic of the SEM and EDX equipment (e.g., power of SEM, power of EDX detector, and the power of the electron beam), which may influence the resolution of data.

In regard to the archaeological sample, we want to stress here that in all EDX histograms presented in the text and interpreted as bone micro residues, we never recorded an overrepresentation of calcite because the Ca/P ratio was always recorded at the fixed and right proportion. Thus, bone residues may not be confused with other calcium mineral when a SEM-EDX analysis is performed. Moreover, bone residues imaged at SEM and OLM always bear well-recognizable morphological features (color, opacity, topography) and, when assisted by the EDX analysis, the doublet of calcium-phosphorous at fixed proportion is always present**.﻿**

The hydroxyapatite is represented on micro FTIR spectra by a shoulder on the low frequency side of the Si-O stretching mode at ~913 cm^-1^ corresponding to the PO_3_= stretching mode of calcium phosphate^28,86^ (apatite). When bone micro residues are analyzed in-situ on stone tools, the most intense peak of hydroxyapatite at around 1030 cm^-1^ is covered by the overlapping Si-O stretching mode of silica. However, the presence of bone produces a broadening at the lower frequency side of this mode and, in addition, a shoulder around 913 cm^-1^. The peak at 913 cm^-1^ is also observed on the spectrum of pure bone.


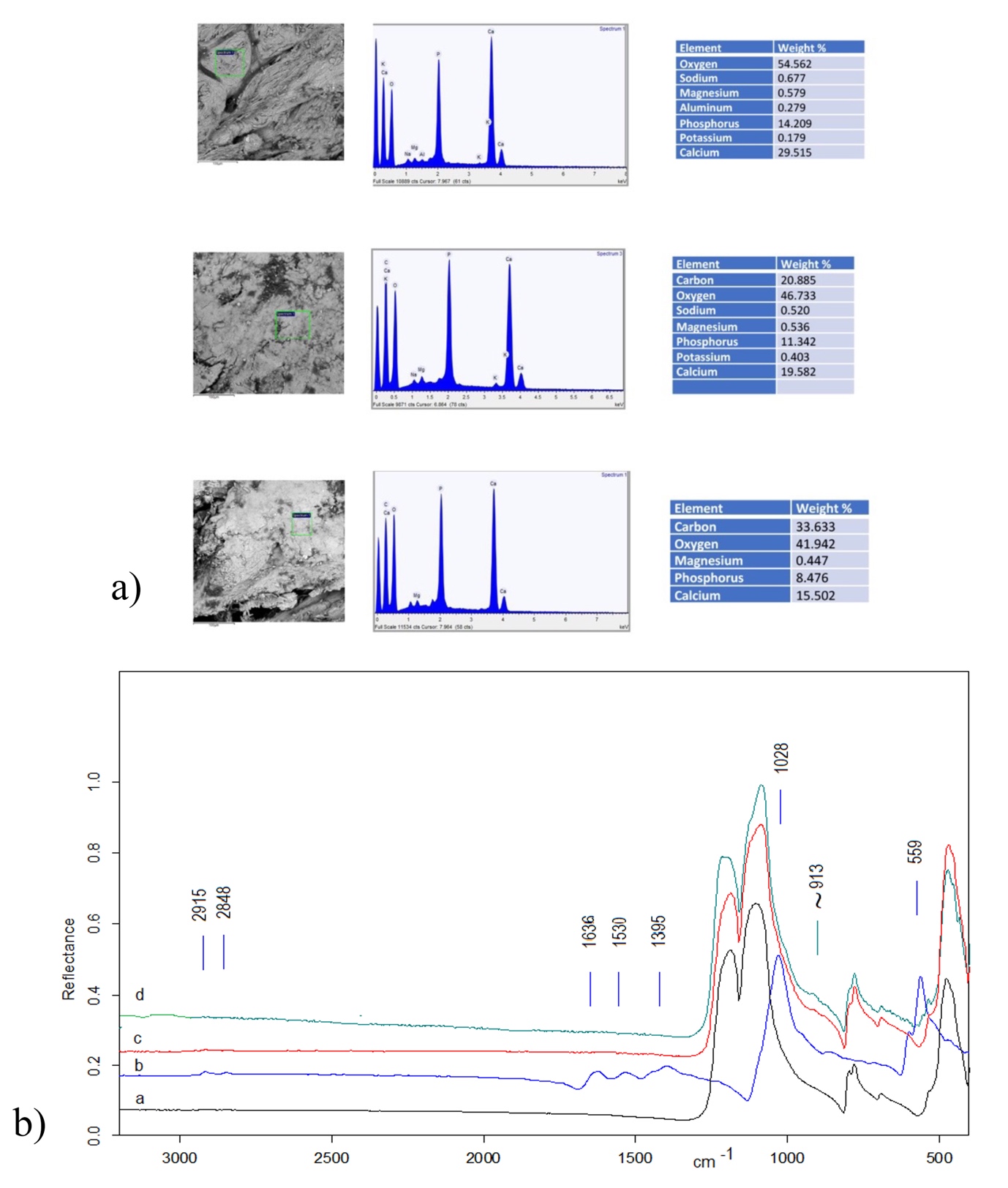


**Fig. S6.** Elemental and chemical analyses of respectively bone tissues and bone residues. a) Three different spots measured through EDX on a modern fragment of a pig bone, showing an average ratio between Ca/P equal to ~1.8. b) ﻿Comparison of micro-FTIR analyses of bone residues: (a) micro-FTIR performed on the archeological fully PFF tool AV14c 71.13-71.10 showing no residues (sample control); (b) ﻿Micro-FTIR performed on pure bone tissues showing the mineral and organic components; (c) Micro-FTIR performed along the active edge on the archeological fully PFF AV14c 71.13-71.10; (d) Micro-FTIR performed on an experimental tool used for processing bone.

# **Supplementary Figures**


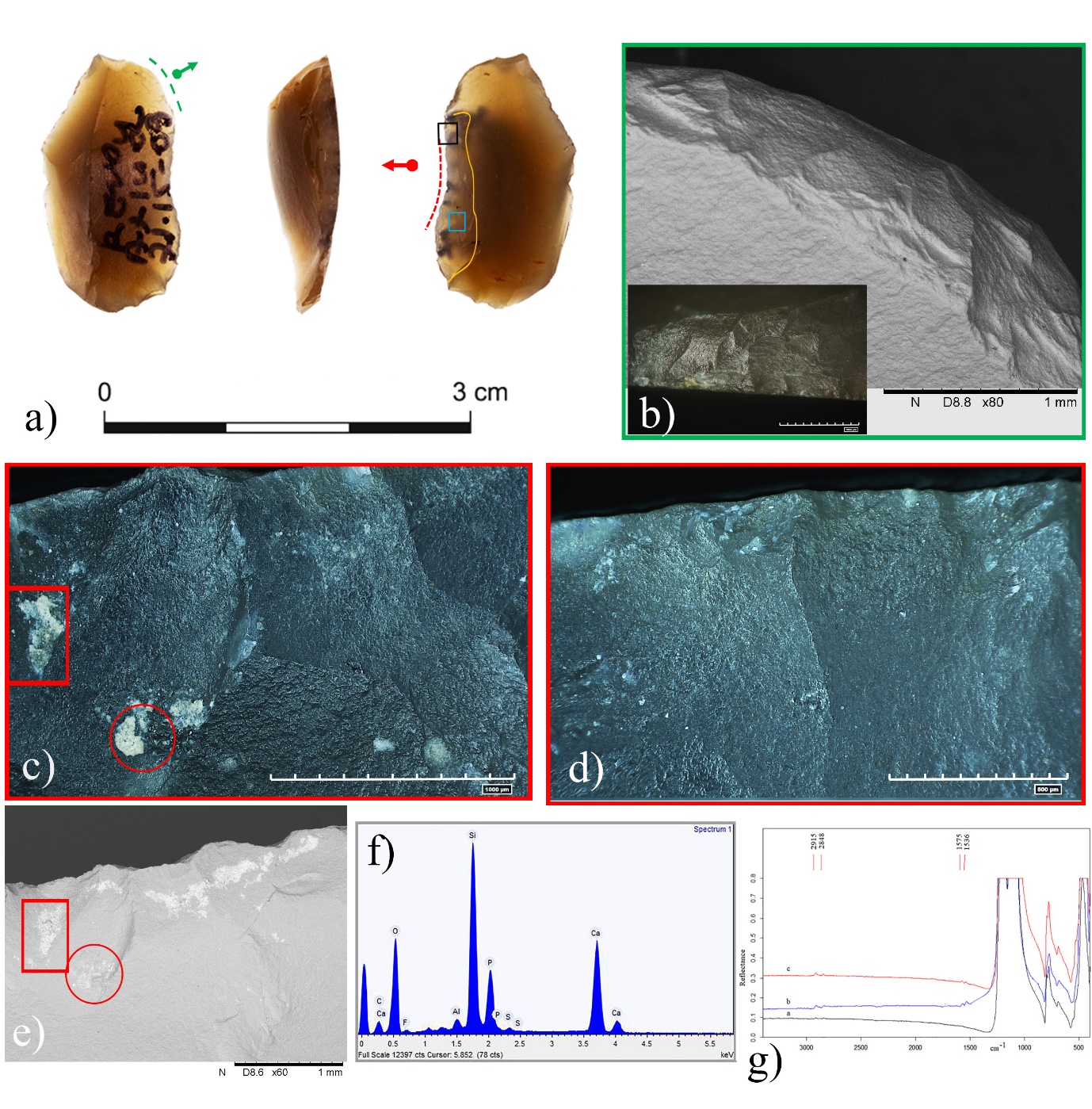


**Fig. S7.** a) Retouched special spall (#7, Ax14a 71.15-09) showing two active edges, one associated with the first use cycle (green dotted line), and the other with the new and last use cycle (red dotted line). The black square marks the location where bone residues were detected. The blue square marks the location where adipocere was detected; b) SEM-micro graph of the steep edge created by the retouch of the proximal edge of the item; c) Macro-residues of bone spread along the item’s new active edge; d) Edge removals on the item’s new active edge; e) SEM-micro graph confirming the bone accumulations along the item’s new active edge; f) Elemental composition of the bone micro-residues; g) Micro-FTIR spectra showing: (a) a spot without residue measured on the archaeological tool, (b) micro-residues of adipocere on an experimental tool used during butchery activity, (c) presence of adipocere detected below the new active edge of the archaeological tool.


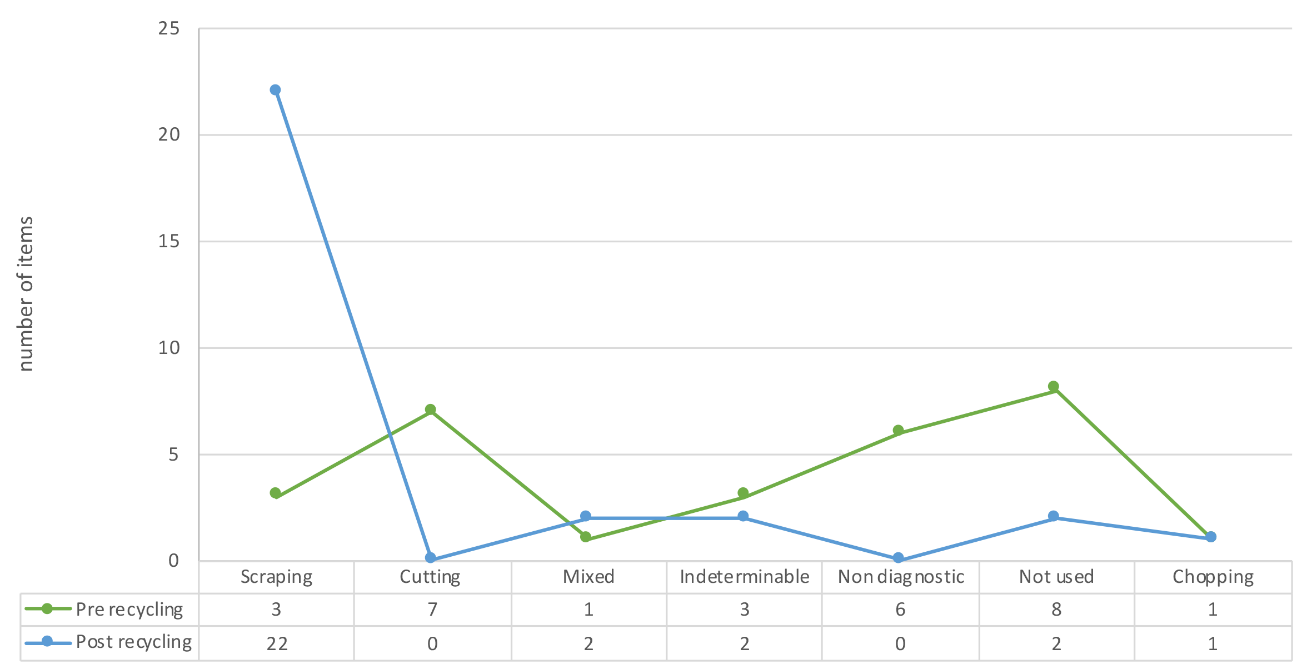


**Fig. S8.** A graph comparing the activities performed using the old and new active edges of all sampled tools that are interpreted as used.

# **Supplementary Tables**

| **Number** | **ID** | **Tool**  **category** | **Use-Wear Description:**  **first life cycle** | **Use-Wear Description: second life cycle** | **Use-wear interpretation: first life cycle** | **Use-wear interpretation: second life cycle** |
| --- | --- | --- | --- | --- | --- | --- |
| 1 | #48  AQ18b+d 71.03-70.99 | Side scraper | **Edge removals**: Oblique irregular halfmoon and hinge scars; **Edge rounding**: Medium | **Edge removals**: Overlapping step scars; **Edge rounding**: Medium | Possibly cutting soft material | Scraping medium material |
| 2 | #6  AW13d 71.14-08 | Side scraper | **Edge removals**: Overlapping step scars; **Edge rounding**: High | **Edge removals**: Absent; **Edge rounding**: Absent | Scraping medium to hard material | Indeterminate |
| 3 | #40  Aw 14d 71.12-10 | Side scraper | **Edge removals**: /  **Edge rounding**: / | **Edge removals**: Snap and overlapping scars; **Edge rounding**: Medium | Not diagnostic | Scraping soft-medium  material |
| 4 | #43  Aq16c 71.15-13 | Side scraper | **Edge removals**: /  **Edge rounding**: / | **Edge removals**: Compression and overlapping scars; **Edge rounding**: Medium to high | Not diagnostic | Scraping medium material |
| 5 | #38  AP14a 71.19-71.14 | Side scraper | **Edge removals**: /  **Edge rounding**: / | **Edge removals**: Perpendicular/oblique feather and hinge scars; **Edge rounding**: Medium to high | No evidence | Scraping on medium material |
| 6 | #39  Ax14b 71.16-09 | Side scraper | **Edge removals**: /  **Edge rounding**: / | **Edge removals**: Close irregular feather and hinge scars; **Edge rounding**: Medium to high | No evidence | Scraping soft-medium material |
| 7 | #45 Ar15a 71.16-06 | Side scraper | **Edge removals**: /  **Edge rounding**: / | **Edge removals**: Overlapping step scars; **Edge rounding**: Medium to high | No evidence | Scraping medium material |
| 8 | #46  AQ16b 71.14-11 | Side scraper | **Edge removals**: /  **Edge rounding**: / | **Edge removals**: Perpendicular overlapping cone feather scars; **Edge rounding**: Medium to high | No evidence | Scraping soft-medium material |
| 9 | #49  AQ17c 71.03-70.01 | Chopping tool | **Edge removals**: Crushing and overlapping step scars; **Edge rounding**: Medium to high | **Edge removals**: Crushing and small overlapping step scars; **Edge rounding**: Medium; **Polish**: Smooth domed on the protruding points | Possibly chopping hard material | Chopping bone |
| 10 | #4  AV14C 71.13-10 | Notch | **Edge removals**: Wide irregular cone hinge scars; **Edge rounding**: medium | **Edge removals**: Close regular feather scars; **Edge rounding:** Low | Cutting soft-medium  material | Scraping soft-medium |
| 11 | #19  AX14d 71.10-07 | Notch | **Edge removals**: /  **Edge rounding: /** | **Edge removals**: Perpendicular overlapping scars; **Edge rounding**: Medium to high | No evidence | Scraping medium to hard material |
| 12 | #30  AV13a71.18-14 | Notch | **Edge removals**: /  **Edge rounding**: **/** | **Edge removals**: Feather and overlapping hinge scars; **Edge rounding**: Medium to high | No evidence | Scraping medium material |
| 13 | #33  AV14c 71.07-05 | Notch | **Edge removals**: /  **Edge rounding**: / | **Edge removals**: Perpendicular overlapping step scars; **Edge rounding**: Medium to high | No evidence | Scraping medium material |
| 14 | #13  AV14 71.13-10 | Notch | **Edge removals**: /  **Edge rounding**: / | **Edge removals**: Perpendicular step and feather scars; **Edge rounding**: Medium | Not diagnostic | Scraping medium  material |
| 15 | #28  AX14b 71.13-09 | Notch | **Edge removals**: /  **Edge rounding**: / | **Edge removals**: Close irregular perpendicular feather scars; **Edge rounding**: Low | Not diagnostic | Scraping soft material |
| 16 | #8  Au16d 71.09-06 | Denticulate | **Edge removals**: /  **Edge rounding**: High | **Edge removals**: Overlapping step scars; **Edge rounding**: Medium | Not diagnostic | Scraping medium  material |
| 17 | #36  AV14c 71.07-05 | Denticulate | **Edge removals**: /  **Edge rounding**: / | **Edge removals**: Close irregular hinge scars; **Edge rounding**: Medium | Not diagnostic | Possibly a scraping activity on medium material |
| 18 | #7  AX14a 71.15-09 | Retouched special spall | **Edge removals**: Overlapping feather and step scars; **Edge rounding**: Medium | **Edge removals**: Overlapping hinge and step scars; **Edge rounding**: Medium | Scraping medium material | Mixed on soft- medium |
| 19 | #32  AV13c 71.09-04 | Retouched flake | **Edge removals**: Slightly oblique step scars; **Edge rounding**: Medium | **Edge removals**: Overlapping irregular hinger scars; **Edge rounding**: Medium; **Polish**: Smooth domed on the protruding points | Scraping soft-medium material | Scraping soft-medium  material |
| 20 | #35  AW13d71.14-08 | Retouched flake | **Edge removals**: Irregular wide scars; **Edge rounding:** medium to high | **Edge removals**: Overlapping hinge and step scars, **Edge rounding**: Medium to high; **Prehension**: Small regular hinge scars | Possibly a cutting activity | Scraping medium-hard material |
| 21 | #17  AV14c 71.07-06 | Retouched flake | **Edge removals**: Oblique step scars; **Edge rounding:** Medium to high | **Edge removals**: /  **Edge rounding**: **/** | Mixed on soft-medium material | Not used |
| 22 | #27  AV16c 71.08-04 | Retouched flake | **Edge removals**: Close regular hinge scars; **Edge rounding**: medium | **Edge removals**: /  **Edge rounding**: | Cutting medium material | Not used |
| 23 | #22  AV13a 71.13-07 | Retouched flake | **Edge removals**: /  **Edge rounding**: / | **Edge removals**: Overlapping step and hinge scars; **Edge rounding**: Medium to high; **Polish**: Smooth domed on the protruding points | Indeterminate | Scraping bone |
| 24 | #31  AV16b 71.07-70.99 | Retouched flake | **Edge removals**: /  **Edge rounding**: **/** | **Edge removals**: Perpendicular overlapping step scars; **Edge rounding**: Medium to high | Indeterminate | Scraping medium to hard material |
| 25 | #14  AW16a 71.10-03 | Retouched flake | **Edge removals**: /  **Edge rounding**: **/** | **Edge removals**: Irregular scars; **Edge rounding**: Medium | No evidence | Indeterminate |
| 26 | #26  AV15a 71.11-08 | Retouched broken flake | **Edge removals**: Close irregular cone hinge scars; **Edge rounding:** Medium | **Edge removals**:  Close irregular feather and hinge scars; **Edge rounding**: Medium | Cutting soft-medium material | Mixed on soft material |
| 27 | #10  AV16d 71.05-00 | Retouched broken flake | **Edge removals**: Close irregular hinge scars; **Edge rounding**: Medium to high | **Edge removals**: Overlapping step scars; **Edge rounding**: Medium to high | Possibly a cutting activity | Scraping medium-hard material |
| 28 | #21  AU13c 71.17-14 | Varia | **Edge removals**: Close irregular feather scars; **Edge rounding**: Medium | **Edge removals**: Perpendicular overlapping step scars; **Edge rounding**: Medium to high | Cutting medium  material | Scraping medium-hard  material |
| 29 | #2  AV16c 71.11-02 | Varia | **Edge removals**: Close regular overlapping hinge scars; **Edge rounding**: Medium to high | **Edge removals**: Overlapping step and hinge scars; **Edge rounding**: high | Indeterminate | Scraping medium material |

**Tab. S1.** List of the fully patinated PPF tools interpreted as used (during their first, second, or both life cycles), and their related use-wear details.

| **Number** | **ID** | **Tool**  **category** | **Residue description** | **FTIR interpretation** | **EDX interpretation** |
| --- | --- | --- | --- | --- | --- |
| 1 | #6  AW13d 71.14-08 | Side scraper | Weak patches of whitish deposit inside a new post-recycling scar | **Old active edge**: None  **New active edge**: None | **Old active edge**: None  **New active edge**: Bone |
| 2 | #39  AX14b 71.16-09 | Side scraper | None | **Old active edge**: None  **New active edge**: Bone | **Old active edge**: None  **New active edge**: None |
| 3 | #45  AR15a 71.16-06 | Side scraper | None | **Old active edge**: None  **New active edge**: Adipocere | Not suitable |
| 4 | #46  AQ16b 71.14-11 | Side scraper | None | **Old active edge**: None  **New active edge**: Bone and adipocere | Not suitable |
| 5 | #48  AQ18b+d 71.03-70.99 | Side scraper | None | **Old active edge**: None  **New active edge**: None | **Old active edge**: None  **New active edge**: None |
| 6 | #4  AV14C 71.13-10 | Notch | ﻿Patches of whitish deposit smeared below the old active edge | **Old active edge**: Bone  **New active edge**: None | **Old active edge**: Bone  **New active edge**: None |
| 7 | #19  AX14d 71.10-07 | Notch | Patches of whitish-yellowish thick deposit smeared below the new active edge | **Old active edge**: None  **New active edge**: Bone | **Old active edge**: None  **New active edge**: Bone |
| 8 | #28  AX14b 71.13-09 | Notch | Thick whitish-yellowish crust deposited within the new retouch scars | **Old active edge**: None  **New active edge**: Bone and adipocere | **Old active edge**: None  **New active edge**: Bone |
| 9 | #30  AV13a71.18-14 | Notch | None | **Old active edge**: None  **New active edge**: Adipocere | **Old active edge**: None  **New active edge**: None |
| 10 | #13  AV14 71.13-10 | Notch | None | **Old active edge**: None  **New active edge**: None | Not suitable |
| 11 | #8  AU16d 71.09-06 | Denticulate | None | **Old active edge**: None  **New active edge**: None | **Old active edge**: None  **New active edge**: None |
| 12 | #7  AX14a 71.15-09 | Retouched special spall | Patches of whitish-yellowish thick deposit smeared below the new active edge | **Old active edge**: None  **New active edge**: Adipocere | **Old active edge**: None  **New active edge**: Bone |
| 13 | #14  AW16a 71.10-03 | Retouched flake | Abundant patches of whitish thick deposit smeared below the new active edge | **Old active edge**: None  **New active edge**: Bone | **Old active edge**: None  **New active edge**: Bone |
| 14 | #35  AW13d71.14-08 | Retouched flake | ﻿Birefringent mass of fat droplets inside the new use-related scars | **Old active edge**: Adipocere  **New active edge**: Adipocere | **Old active edge**: None  **New active edge**: None |
| 15 | #22  AV13a 71.13-07 | Retouched flake | None | **Old active edge**: None  **New active edge**: Adipocere | **Old active edge**: None  **New active edge**: Bone |
| 16 | #17  AV14c 71.07-06 | Retouched flake | None | **Old active edge**: None  **New active edge**: None | **Old active edge**: None  **New active edge**: None |
| 17 | #31  AV16b 71.07-70.99 | Retouched flake | None | **Old active edge**: None  **New active edge**: None | **Old active edge**: None  **New active edge**: None |
| 18 | #32  AV13c 71.09-04 | Retouched flake | None | **Old active edge**: None  **New active edge**: None | **Old active edge**: None  **New active edge**: None |
| 19 | #10  AV16d 71.05-00 | Retouched broken flake | None | **Old active edge**: None  **New active edge**: Weak adipocere | **Old active edge**: None  **New active edge**: None |
| 20 | #26  AV15a 71.11-08 | Retouched broken flake | None | **Old active edge**: None  **New active edge**: None | **Old active edge**: None  **New active edge**: None |
| 21 | #21  AU13c 71.17-14 | Varia | Patches of whitish deposit smeared below the old active edge | **Old active edge**: None  **New active edge**: None | **Old active edge**: Bone  **New active edge**: None |
| 22 | #2  AV16c 71.11-02 | Varia | None | **Old active edge**: None  **New active edge**: Adipocere | **Old active edge**: None  **New active edge**: None |

**Tab. S2.** List of the fully patinated PPF tools interpreted as used (during their first, second, or both cycles) and bearing evidence of residues identified through optical observations, FTIR and SEM-EDX analyses.

| Area C (layer C3) | C3-East | | | C3-West | | | C3-total | | |
| --- | --- | --- | --- | --- | --- | --- | --- | --- | --- |
| Categories | **n.** | **% of débitage and shaped items** | **% of total assemblage** | **n.** | **% of débitage and shaped items** | **% of total assemblage** | **n.** | **% of débitage and shaped items** | **% of total assemblage** |
| Primary element flake  (PE flake) | 844 | 6.3% | 3.0% | 1553 | 11.6% | 5.5% | 2397 | 18.0% | 8.4% |
| Primary element blade  (PE blade) | 25 | 0.2% | 0.1% | 15 | 0.1% | 0.1% | 40 | 0.3% | 0.1% |
| Flakes | 1596 | 12.0% | 5.6% | 2138 | 16.0% | 7.5% | 3734 | 28.0% | 13.1% |
| Lipped flakes | 1 | 0.0% | 0.0% | 29 | 0.2% | 0.1% | 30 | 0.2% | 0.1% |
| Blades | 34 | 0.3% | 0.1% | 53 | 0.4% | 0.2% | 87 | 0.7% | 0.3% |
| Core trimming elements (CTE) | 485 | 3.6% | 1.7% | 619 | 4.6% | 2.2% | 1104 | 8.3% | 3.9% |
| Cores | 488 | 3.7% | 1.7% | 835 | 6.3% | 2.9% | 1323 | 9.9% | 4.7% |
| Flaked pebbles | 30 | 0.2% | 0.1% | 97 | 0.7% | 0.3% | 127 | 1.0% | 0.4% |
| Core-on-flakes (COF) | 342 | 2.6% | 1.2% | 602 | 4.5% | 2.1% | 944 | 7.1% | 3.3% |
| Shaped items (Tools) | 960 | 7.2% | 3.4% | 1581 | 11.9% | 5.6% | 2546 | 19.1% | 9.0% |
| Recycling products | 320 | 2.4% | 1.1% | 388 | 2.9% | 1.4% | 708 | 5.3% | 2.5% |
| Special waste (sp. sp.) | 194 | 1.5% | 0.7% | 107 | 0.8% | 0.4% | 301 | 2.3% | 1.1% |
| Total débitage  and shaped items | **5319** | **100.0%** | **18.7%** | **8017** | **100.0%** | **28.2%** | **13341** | **100.0%** | **46.9%** |
| Broken flakes | 1855 |  | 6.5% | 2862 |  | 10.1% | 4717 |  | 16.6% |
| Chunks | 615 |  | 2.2% | 596 |  | 2.1% | 1211 |  | 4.3% |
| Chips | 4769 |  | 16.8% | 2483 |  | 8.7% | 7252 |  | 25.5% |
| Micro flakes | 923 |  | 3.2% | 798 |  | 2.8% | 1721 |  | 6.1% |
| Untreated nodules |  |  | 0.0% |  |  | 0.0% | 202 |  | 0.7% |
| Total | **8162** |  | **28.7%** | **14756** |  | **51.9%** | **28444** |  | **100.0%** |

**Tab. S3.** General breakdown of the lithic assemblage of layer C3 (East + West).
